# Adipocyte Fatty Acid Binding Protein Promotes the Onset and Progression of Liver Fibrosis via Mediating the Crosstalk between Liver Sinusoidal Endothelial Cells and Hepatic Stellate Cells

**DOI:** 10.1002/advs.202003721

**Published:** 2021-03-27

**Authors:** Xiaoping Wu, Lingling Shu, Zixuan Zhang, Jingjing Li, Jiuyu Zong, Lai Yee Cheong, Dewei Ye, Karen S. L. Lam, Erfei Song, Cunchuan Wang, Aimin Xu, Ruby L. C. Hoo

**Affiliations:** ^1^ State Key Laboratory of Pharmaceutical Biotechnology LKS Faculty of Medicine the University of Hong Kong Hong Kong 999077 China; ^2^ Department of Pharmacology and Pharmacy LKS Faculty of Medicine the University of Hong Kong Hong Kong 999077 China; ^3^ Department of Medicine LKS Faculty of Medicine the University of Hong Kong Hong Kong 999077 China; ^4^ State Key Laboratory of Oncology in South China Collaborative Innovation Center for Cancer Medicine Sun Yat‐sen University Cancer Center Guangzhou 510060 China; ^5^ Joint Laboratory of Guangdong and Hong Kong on Metabolic Diseases Guangdong Pharmaceutical University Guangzhou 510000 China; ^6^ Department of Metabolic and Bariatric Surgery The First Affiliated Hospital of Jinan University Guangzhou Guangdong 510630 China; ^7^ HKU‐Shenzhen Institute of Research and Innovation (HKU‐SIRI) Shenzhen 518057 China

**Keywords:** A‐FABP, hepatic stellate cells, liver fibrosis, liver sinusoidal endothelial cells, TGF*β*1

## Abstract

Development of liver fibrosis results in drastic changes in the liver microenvironment, which in turn accelerates disease progression. Although the pathological function of various hepatic cells in fibrogenesis is identified, the crosstalk between them remains obscure. The present study demonstrates that hepatic expression of adipocyte fatty acid binding protein (A‐FABP) is induced especially in the liver sinusoidal endothelial cells (LSECs) in mice after bile duct ligation (BDL). Genetic ablation and pharmacological inhibition of A‐FABP attenuate BDL‐ or carbon tetrachloride‐induced liver fibrosis in mice associating with reduced collagen accumulation, LSEC capillarization, and hepatic stellate cell (HSC) activation. Mechanistically, elevated A‐FABP promotes LSEC capillarization by activating Hedgehog signaling, thus impairs the gatekeeper function of LSEC on HSC activation. LSEC‐derived A‐FABP also acts on HSCs in paracrine manner to potentiate the transactivation of transforming growth factor *β*1 (TGF*β*1) by activating c‐Jun N‐terminal kinase (JNK)/c‐Jun signaling. Elevated TGF*β*1 subsequently exaggerates liver fibrosis. These findings uncover a novel pathological mechanism of liver fibrosis in which LSEC‐derived A‐FABP is a key regulator modulating the onset and progression of the disease. Targeting A‐FABP may represent a potential approach against liver fibrosis.

## Graphical abstract

Chronic liver injury upregulates adipocyte fatty acid binding protein (A‐FABP) in liver sinusoidal endothelial cells (LSECs). A‐FABP potentiates LSEC capillarization through activating Hh signaling, thus initiating hepatic stellate cell (HSC) activation. Moreover, LSEC‐derived A‐FABP stimulates TGF*β*1 expression in HSCs through activating JNK/c‐Jun signaling. Enhanced TGF*β*1 further perpetuates hepatic fibrogenesis.

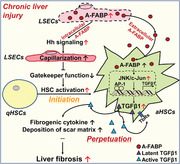

## 1 Introduction

Liver fibrosis is characterized by the excessive deposition of extracellular matrix (ECM), which leads to hepatic architectural distortion resulting in pathophysiologic damage to the organ.^[^ ^1^ ^]^ It is the advanced stage of chronic liver disease which can proceed to cirrhosis and may ultimately result in hepatocellular carcinoma^[^ ^1^ ^]^ while it is also the last reversible stage.^[^ ^2^ ^]^ Various hepatic cells were shown to modulate the local microenvironment contributing to the liver fibrosis^[^ ^3^ ^]^ while no FDA‐approved medication is available suggesting the interactions between cells and the underlying mechanism are still poorly understood.

Viral infection, obesity‐related nonalcoholic fatty liver disease (NAFLD) and nonalcoholic steatohepatitis (NASH), chronic alcohol abuse, autoimmune disorder, and cholestatic disorder are the etiologies of liver fibrosis.^[^ ^1^ ^]^ Capillarization of liver sinusoidal endothelial cells (LSECs) and activation of hepatic stellate cells (HSCs) are the key events in the kickoff and progression of the disease. Upon prolonged damage or stimulation, LSECs undergo phenotypic changes with the loss of fenestrae (capillarization) and exhibit reduced gatekeeper function on HSC activation.^[^ ^4^ ^]^ Quiescent HSCs (qHSCs) are trans‐differentiated into myofibroblast‐like activated HSCs (aHSCs), which are the major producers of ECM proteins such as fibril‐forming collagens, elastin, and fibronectin.^[^ ^1^ ^]^ HSC activation consists of two phases: initiation and perpetuation. Initiation majorly results from the paracrine stimulation of neighboring cells such as damaged hepatocytes, capillarized LSECs, and activated inflammatory cells. Perpetuation then amplifies the activated phenotypes of HSCs to increase ECM accumulation.^[^ ^5^ ^]^ Among factors in autocrine or paracrine loops, transforming growth factor‐beta1 (TGF*β*1) is a key regulator of the expression and secretion of ECM proteins from HSCs, while suppresses ECM degradation by increasing the production of tissue inhibitor of metalloproteinase‐1 (TIMP‐1).^[^ ^6^ ^]^ However, the factors that promote LSEC capillarization, HSC activation, and TGF*β*1 expression remain largely unknown.

Adipokines such as leptin, adiponectin, and resistin are implicated in the progression of liver diseases.^[^ ^7^ ^]^ Adipocyte fatty acid‐binding protein (A‐FABP), also known as aP2 and FABP4, is an adipokine that primarily acts as a lipid chaperone to transport fatty acids between cellular organelles or in the circulation to regulate lipid metabolism.^[^ ^8^ ^]^ It is abundantly expressed in adipocytes while can also be expressed in endothelial cells, macrophages, and dendritic cells.^[^ ^8^, ^9^ ^]^ Treatment with A‐FABP selective inhibitor BMS309403 alleviated high fat diet‐induced NASH in mice.^[^ ^10^ ^]^ Increased hepatic A‐FABP expression is detected in patients with liver cirrhosis correlating with clinical outcomes^[^ ^11^ ^]^ and is observed in patients with hepatocellular carcinoma with multiple metabolic risk factors.^[^ ^12^ ^]^ Circulating level of A‐FABP increases from steatosis to nonalcoholic steatohepatitis and is positively correlated with the inflammatory grade and fibrotic stage in patients with NAFLD.^[^ ^13^ ^]^ However, whether A‐FABP possesses a direct pathophysiological role in hepatic fibrogenesis regardless to its effect on metabolic syndrome has never been explored.

In the present study, A‐FABP knockout (A‐FABP KO) mice and their wild‐type (WT) littermates were subjected to cholestasis‐ or hepatotoxin‐induced liver fibrosis by surgical ligation of the common bile duct (BDL) or chronic administration of carbon tetrachloride (CCl_4_)^[^ ^14^ ^]^ to determine the exact role and the molecular mechanism that A‐FABP involved in hepatic fibrogenesis. The therapeutic potential of BMS309403 on liver fibrosis was also explored.

## 2 Results

### 2.1 A‐FABP Deficiency Ameliorates Liver Fibrosis in Mice

**Figure 1:**
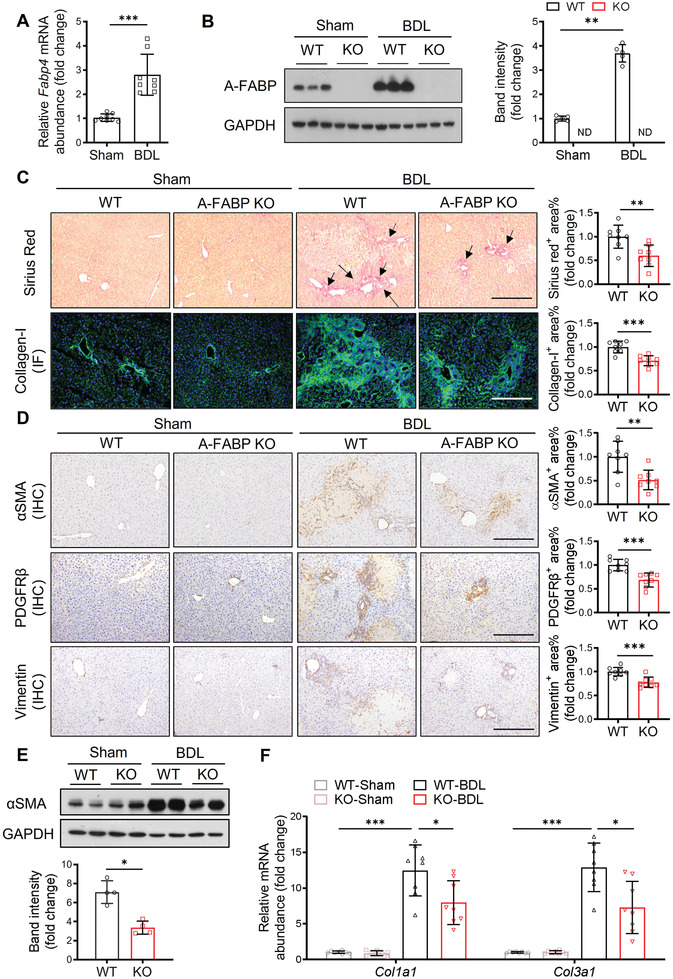
A‐FABP deficiency ameliorates BDL‐induced liver fibrosis in mice. A‐FABP KO mice and their WT littermates were subjected to BDL or sham operation for two weeks. A) Relative mRNA abundance of hepatic *Fabp4* in WT mice (*n* = 8). B) Representative immunoblots of the hepatic expression of A‐FABP and GAPDH in mice and the band intensity of A‐FABP relative to GAPDH (*n* = 5). Representative images of C) Sirius red staining and immunofluorescence (IF) staining of collagen‐I, and D) immunohistochemistry (IHC) staining of *α*SMA, PDGFR*β*, and vimentin of mouse liver sections (100 ×, scale bar 250 µm) (*n* = 8). Black arrow in panel C indicates the mature collagen stained by Sirius red. Right panels are the densitometry analysis of the positive area of Sirius red, collagen‐I, *α*SMA, PDGFR*β*, and vimentin of mice in BDL group, respectively (*n* = 8). E) Representative immunoblots of the hepatic expression of *α*SMA and GAPDH in mice. Lower panel is the band intensity of *α*SMA of mice in BDL group relative to GAPDH (*n* = 4). F) Relative mRNA abundance of hepatic *Col1a1* and *Col3a1* (*n* = 8). Data are presented as mean ± SD. *P < 0.05, **P < 0.01, ***P < 0.001. Mann‐Whitney *U* test was used in (A) and (E). Unpaired Student's *t* test was used in (B), (C), and (D). Two‐way ANOVA followed by Tukey's test was used in (F).

To determine the role of A‐FABP in liver fibrosis, A‐FABP KO male mice and their WT littermates were subjected to BDL or CCl_4_ treatment to induce fibrosis. After BDL, the hepatic mRNA and protein levels of A‐FABP (encoded by *Fabp4* gene) were significantly induced in WT mice and the absence of A‐FABP in the A‐FABP KO mice was confirmed (**Figure** **1A**,B). Hepatic A‐FABP levels were also significantly elevated in CCl_4_‐exposed mice comparing to their relative controls (Figure S1A,B, Supporting Information).

BDL‐induced hepatic accumulation of mature collagen fibers,^[^ ^1^ ^]^ type‐I collagen (collagen‐I)^[^ ^1^ ^]^ (Figure 1C), and HSC activation as indicated by the expression of alpha‐smooth muscle actin (*α*SMA) (Figure 1D,E),^[^ ^1^ ^]^ platelet‐derived growth factor receptor‐beta (PDGFR*β*),^[^ ^15^ ^]^ and vimentin^[^ ^16^ ^]^(Figure 1D) were significantly attenuated in A‐FABP KO mice when compared to the WT littermates. Consistently, the mRNA expression of fibrogenesis markers including collagen‐I*α*1 (encoded by *Col1a1*) and collagen‐III*α*1 (encoded by *Col3a1)* was significantly decreased in A‐FABP KO mice comparing to WT mice (Figure 1F). In female mice after BDL, the induction of hepatic A‐FABP in WT mice and the attenuation of collagen accumulation in A‐FABP KO mouse liver comparing to their control mice were also observed (Figure S2, Supporting Information). Moreover, CCl_4_‐induced WT mice exhibited bridging fibrosis^[^ ^1^ ^]^ as indicated by the collagen bands extend across lobules between portal areas was not developed in A‐FABP KO mice (Figure S1C, Supporting Information). These data implicated that A‐FABP plays a pathological role in liver fibrosis.

### 2.2 Liver Sinusoidal Endothelial cell (LSEC) Is the Major Hepatocellular Source of A‐FABP in Fibrotic Liver

**Figure 2:**
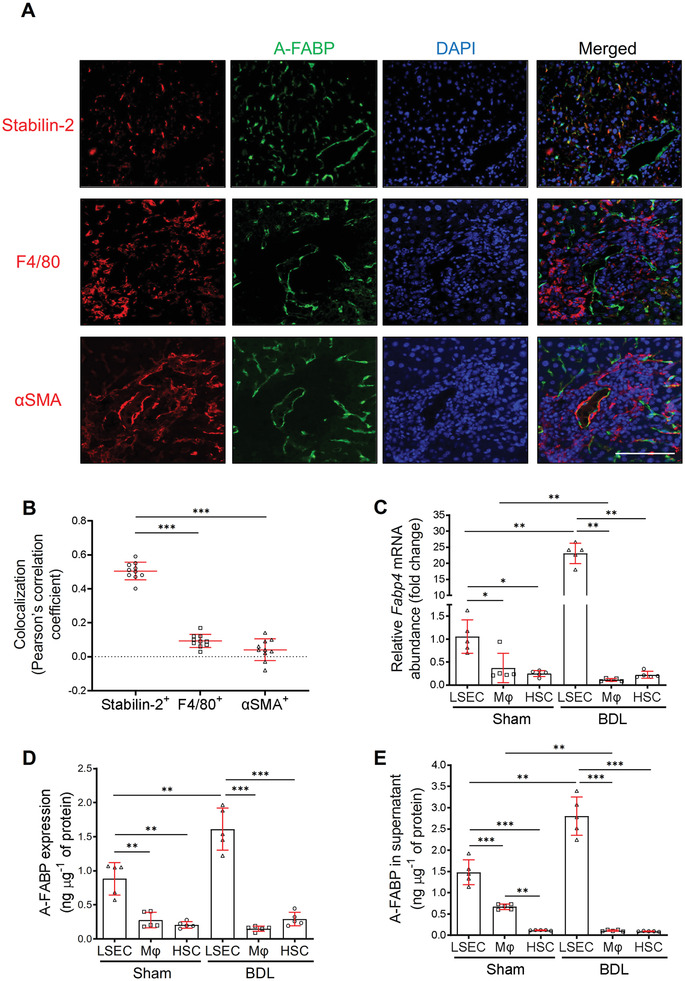
LSEC is the major hepatocellular source of A‐FABP in BDL‐induced liver fibrosis. WT mice were subjected to sham operation or BDL for 2 weeks. A) Representative images of immunofluorescence co‐staining of A‐FABP with stabilin‐2, F4/80, or *α*SMA of liver sections from BDL‐induced mice (scale bar 100 µm) (*n* = 10). B) Colocalization analysis of A‐FABP^+^ cells with stabilin‐2^+^, F4/80^+^, or *α*SMA^+^ cells in co‐staining of panel A, data presented as Pearson's correlation coefficient (PCC) (*n* = 10). LSECs, macrophages (M*φ*), and HSCs were isolated from WT mice after BDL or sham operation. C) Relative mRNA abundance of *Fabp4* in various cell types (*n* = 5). D) The concentration of A‐FABP in various cell types normalized with the total protein content of cells (*n* = 5). E) LSECs, macrophages, and HSCs were isolated from WT mice with BDL or sham operation and cultured for 12 hours. The concentration of A‐FABP in conditioned media (CM) normalized with total protein content in cell lysate (*n* = 5). Data are presented as mean ± SD. *P < 0.05, **P < 0.01, ***P < 0.001. One‐way ANOVA followed by Tukey's test was used in (B). Unpaired Student's *t* test or Mann‐Whitney *U* test was used in (C), (D), and (E).

Murine hepatic A‐FABP is mainly expressed in the nonparenchymal cell fraction.^[^ ^10^ ^]^ We next determined the hepatic cellular source of A‐FABP in response to BDL‐induced liver fibrosis. Upon BDL, the expression of A‐FABP was mainly colocalized with stabilin‐2^+^ cells (LSECs)^[^ ^4^, ^17^ ^]^ with the Pearson's correlation coefficient^[^ ^18^ ^]^ (PCC) = 0.504 ± 0.052. A‐FABP also colocalized with F4/80^+^ cells (Kupffer cells and infiltrated macrophages)^[^ ^19^ ^]^ and *α*SMA^+^ cells (activated HSCs and vascular smooth muscle cells)^[^ ^20^ ^]^ with PCC = 0.093 ± 0.038 and 0.041 ± 0.063, respectively, which were significantly lower than that with stabilin‐2^+^ cells (**Figure** **2A**,B).

Primary LSECs (stabilin‐2^+^‐cd11b^−^),^[^ ^21^ ^]^ macrophages (cd11b^+^‐F4/80^+^),^[^ ^21^ ^]^ and HSCs (retinoid autofluorescence^+^)^[^ ^22^ ^]^ were isolated from liver of WT mice after BDL or sham operation with purity higher than 95% (Figure S3, Supporting Information). In sham‐operated mice, the A‐FABP mRNA and protein expression were detected in all the isolated hepatic cell types and were most abundant in LSECs (Figure 2C, D). In response to BDL, the A‐FABP expression in LSECs was further induced significantly while the mRNA expression in macrophages and HSCs was declined and unchanged, respectively, and no difference in protein expression between BDL and sham‐operated mice was observed (Figure 2C,D). Furthermore, the A‐FABP level in the conditioned media (CM) of LSECs isolated from BDL‐induced mice was significantly increased while those from macrophages and HSCs were decreased or not changed, respectively, when compared to those CM of LSECs from the sham‐operated mice (Figure 2E). Notably, the amount of A‐FABP in the CM of LSECs was higher than that in its cell lysate (Figure 2D,E). These results indicated that LSEC is the major cellular source of hepatic A‐FABP in liver fibrosis, which can be secreted into the microenvironment contributing to liver fibrosis.

### 2.3 A‐FABP Promotes the Onset of HSC Activation via Impairing the Gatekeeper Function of LSECs

**Figure 3:**
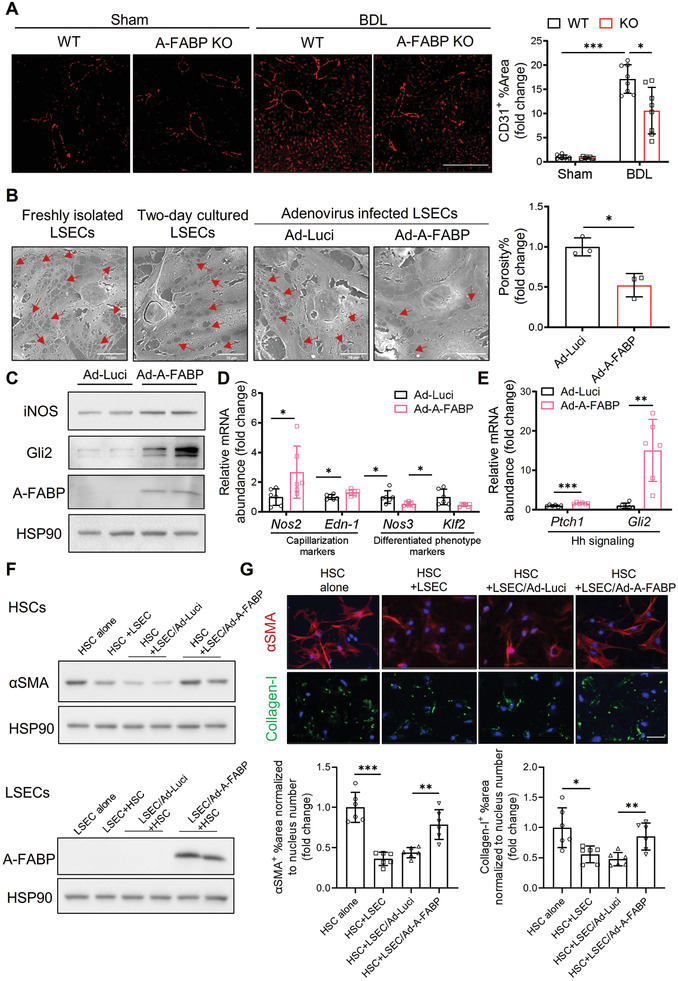
A‐FABP potentiates LSEC capillarization leading to initiation of HSC activation. A‐FABP KO mice and their WT littermates were subjected to BDL or sham operation for two weeks. A) Representative images of immunofluorescent staining of CD31 of mouse liver sections (scale bar 200 µm) (*n* = 8). Right panel is the densitometry analysis of CD31 positive area (*n* = 8). B–E) Primary A‐FABP KO LSECs were infected with adenovirus‐overexpressing luciferase (Ad‐Luci) or adenovirus‐overexpressing A‐FABP (Ad‐A‐FABP) (50 MOI) for 2 d after isolation. B) Phenotypic change of freshly isolated LSECs (3 hours after isolation), two‐day cultured LSECs, and adenovirus‐infected LSECs were examined by scanning electron microscopy (scale bar 10 µm). Red arrows indicate the fenestrae and sieve plates. Right panel is the quantification of the percentage of fenestrae to total surface area and represented as percentage of porosity (*n* = 3). C) Representative immunoblots of A‐FABP, iNOS, Gli2, and HSP90 in LSECs. D,E) mRNA abundance of D) LSEC capillarization‐related markers (*Nos2*, *Edn‐1*) and differentiated phenotype‐related markers (*Nos3* and *Klf2*) and E) Hh signaling target genes (*Ptch1* and *Gli2*). F,G) A‐FABP KO HSCs were cultured alone (HSC alone), cocultured with A‐FABP KO LSECs (HSC+LSEC), cocultured with A‐FABP KO LSECs infected with Ad‐Luci (HSC+LSEC/Ad‐Luci), or cocultured with A‐FABP KO LSECs infected with Ad‐A‐FABP (HSC+LSEC/Ad‐A‐FABP), respectively. F) Representative immunoblots of *α*SMA and HSP90 in HSCs (upper), and A‐FABP and HSP90 in LSECs (lower). G) Representative images of immunofluorescent staining of *α*SMA and collagen‐I in HSCs (scale bar 50 µm). Lower panels are the densitometry analysis of *α*SMA and collagen‐I positive area normalized to nucleus number and presented as fold change (*n* = 6). Data are presented as mean ± SD. *P < 0.05, **P < 0.01, ***P < 0.001. Two‐way ANOVA followed by Tukey's test was used in (A). Unpaired Student's *t* test or Mann‐Whitney *U* test was used in (B), (D), (E) and (G).

LSECs are known as a determinant of liver fibrosis.^[^ ^4^ ^]^ Upon chronic stimulation of damage factors, differentiated LSECs undergo capillarization which leads to the loss of its gatekeeper function for HSC activation.^[^ ^4^ ^]^ As A‐FABP is mainly elevated in LSECs in liver fibrosis (Figure 2), we determined whether A‐FABP modulates LSEC capillarization. After BDL, capillarization of LSECs was significantly induced in WT mice as indicated by the increased expression of CD31^[^ ^23^ ^]^ in hepatic lobule (**Figure** **3A**). However, this induction was attenuated in A‐FABP KO mice (Figure 3A). BDL‐induced mRNA expression of LSEC capillarization markers^[^ ^24^ ^]^ including von Willebrand factor (vWF, encoded by *Vwf*), endothelin‐1 (ET‐1, encoded by *Edn‐1*), and nitric oxide synthase (iNOS, encoded by *Nos2*) were also significantly attenuated in LSECs of A‐FABP KO mice when compared to those of WT mice (Figure S4, Supporting Information).

Primary A‐FABP KO LSECs were isolated and followed by purity check using its unique endocytic capacity of ac‐LDL uptake and counter‐staining for macrophages (F4/80^+^). LSECs were defined as Ac‐LDL^+^‐F4/80^−^ cells^[^ ^21^ ^]^ (Figure S5A,B, Supporting Information). LSECs (purity > 95%) were subsequently infected with adenovirus‐overexpressing‐A‐FABP (Ad‐A‐FABP) or ‐luciferase (Ad‐Luci) as control. The phenotypic change of LSECs was assessed using electronic microscopy. Previous study revealed that numerous fenestrae and sieve plates were maintained in day 1 cultured LSECs and were gradually decreased in day 2 and 3 cultured LSECs. In day 4 and day 5 cultured LSECs, fenestrae could only be observed occasionally.^[^ ^24^ ^]^ In line with previous findings,^[^ ^24^ ^]^ freshly isolated A‐FABP KO LSECs exhibited numerous fenestrae and sieve plates which were gradually reduced after 2 day culture suggesting spontaneous capillarization in A‐FABP KO LSECs while they are not fully capillarized. A‐FABP KO LSECs with luciferase overexpression showed a comparable number of fenestrae and sieve plates as that of two‐day cultured A‐FABP KO LSECs while A‐FABP overexpression further decreased the number of fenestrae and sieves plates comparing to its relative controls (Figure 3B). Consistently, overexpression of A‐FABP in A‐FABP KO LSECs induced iNOS protein expression (Figure 3C) as well as the mRNA expression of LSECs capillarization markers^[^ ^24^ ^]^ *Nos2* and *Edn‐1* while downregulated those of endothelial nitric oxide synthase (eNOS, encoded by *Nos3*)^[^ ^23^ ^]^ and Kruppel‐like factor 2 (KLF2, encoded by *Klf2*),^[^ ^25^ ^]^ the essential factors for maintaining the differentiated phenotype of LSECs (Figure 3D). LSEC capillarization is mediated by the activation of Hedgehog (Hh) signaling.^[^ ^24^ ^]^ Upon A‐FABP overexpression, the mRNA expression of Hh signaling downstream targets including protein patched homolog 1 (Ptch1, encoded by *Ptch1*) and Zinc finger protein Gli2 (Gli2, encoded by *Gli2*) (Figure 3E) and its protein (Figure 3C), were significantly elevated. Taken together, these results indicated that A‐FABP potentiates LSEC capillarization via activating Hh signaling.

Previous in vitro coculture study^[^ ^26^ ^]^ demonstrated that differentiated LSECs prevents

HSC activation, we next determined whether A‐FABP overexpression in LSEC impairs its gatekeeper function on HSC activation. The purity and identity of LSECs and HSCs subjected to the in vitro experiment were confirmed (Figure S5, Supporting Information). Consistent with previous study,^[^ ^26^ ^]^ coculture of primary HSCs with LSECs suppressed the expression of *α*SMA and collagen‐I in HSCs (Figure 3F,G). When HSCs were cocultured with LSECs overexpressing A‐FABP, the expression of *α*SMA and collagen‐I was significantly induced when compared to those cocultured with LSECs infected with adenovirus overexpressing luciferase (Ad‐Luci) (Figure 3F,G). These findings implicated that the elevation of A‐FABP in LSECs stimulates LSEC capillarization thus impairs its gatekeeper function on HSC activation.

### 2.4 A‐FABP Deficiency Attenuates BDL‐Induced Activation of TGF*β*1/Smad Signaling

**Figure 4:**
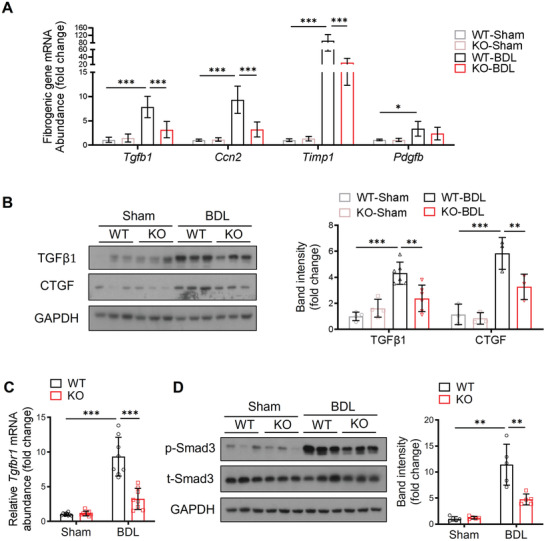
A‐FABP deficiency attenuates BDL‐induced activation of TGF*β*1/Smad signaling in the liver of mice. A‐FABP KO mice and their WT littermates were subjected to BDL or sham operation for two weeks (*n* = 8). A) Relative mRNA abundance of hepatic *Tgfb1, Ccn2, Timp1*, and *Pdgfb* (*n* = 8). B) Representative immunoblots of hepatic TGF*β*1, CTGF, and GAPDH and the band intensities of various proteins relative to GAPDH (*n* = 3–6). C) Relative mRNA abundance of *Tgfbr1* in mouse liver (*n* = 8). D) Representative immunoblots of hepatic p‐Smad3 (Ser 423/425), t‐Smad3, and GAPDH and the band intensities of p‐Smad3 relative to t‐Smad‐3 (*n* = 5). Data are presented as mean ± SD. *P < 0.05, **P < 0.01, ***P < 0.001. Two‐way ANOVA followed by Tukey's test was used in (A), (B), (C), and (D).

To further determine the linking factors between A‐FABP and liver fibrosis, the hepatic expression of fibrogenic cytokines was examined.^[^ ^6^, ^27^ ^]^ BDL‐induced mRNA levels of TGF*β*1 (encoded by*Tgfb1*), connective tissue growth factor (CTGF, encoded by *Cnn2*), and TIMP‐1 (encoded by *Timp1*) were significantly attenuated in A‐FABP KO mice, while that of platelet‐derived growth factor subunit B (PDGFB, encoded by *Pdgfb*) was not altered when compared to those in WT mice (**Figure** **4A**). Among these cytokines, TGF*β* exerts a pivotal role in hepatic fibrogenesis and modulates the expression of CTGF and TIMP‐1.^[^ ^6^ ^]^ TGF*β*1 modulates the transcription of its target genes by binding to the TGF*β* receptors (T*β*Rs) and the subsequent activation of Smads signaling.^[^ ^6^ ^]^ The expression of T*β*R‐I is also regulated in a ligand‐dependent manner.^[^ ^28^ ^]^ We showed that BDL induced the protein expression of TGF*β*1 and its downstream target CTGF in both WT and A‐FABP KO mice but the magnitude of induction in A‐FABP KO mice was significantly lower (Figure 4B). Attenuated expression of TGF*β*1 in A‐FABP KO mice was associated with reduced hepatic mRNA expression of T*β*R‐I (encoded by *Tgfbr1*) (Figure 4C). A‐FABP deficiency also suppressed CCl_4_‐induced hepatic expression of TGF*β*1 in mice (Figure S6, Supporting Information). Consistently, the activation of Smads signaling, as indicated by Smad3 phosphorylation, was attenuated in the A‐FABP KO mice comparing to the WT mice (Figure 4D). These data implicated that A‐FABP modulates the hepatic expression of TGF*β*1 which further potentiates its downstream T*β*R‐I/Smad3 signaling contributing to liver fibrosis.

### 2.5 LSEC‐Derived A‐FABP Promotes the Perpetuation of HSC Activation via Stimulating TGF*β*1 Expression in HSCs

**Figure 5:**
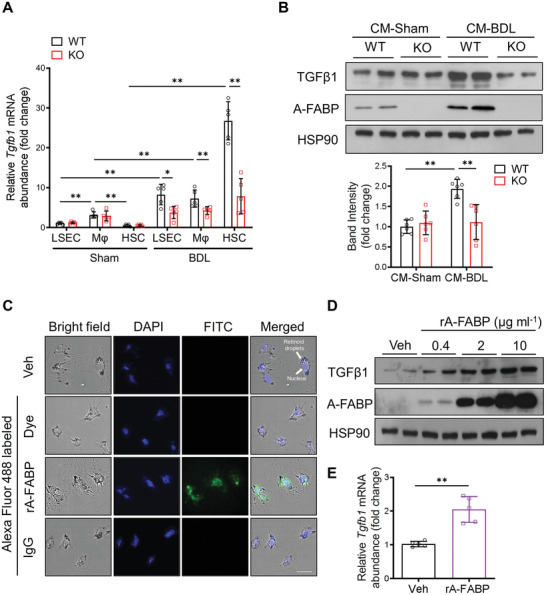
LSEC‐derived A‐FABP exaggerates TGF*β*1 expression in activated HSCs. A) LSECs, macrophages (M*φ*), and HSCs were isolated from WT or A‐FABP KO mice after BDL or sham operation for two weeks. Relative mRNA abundance of *Tgfb1* in various cell types (*n* = 5). B) Representative immunoblots of TGF*β*1, A‐FABP, and HSP90 of culture‐activated A‐FABP KO HSCs incubated with conditioned media (CM) of LSECs isolated from BDL‐ or sham‐operated mice for 24 hours. Lower panel is the band intensities of TGF*β*1 relative to HSP90 (*n* = 6). C) The representative images of culture‐activated A‐FABP KO HSCs treated with vehicle (veh, PBS), Alexa Fluor 488 dye, Alexa Fluor 488 labeled rA‐FABP (2 µg mL^−1^), or Alexa Fluor 488 labeled IgG (2 µg mL^−1^) for 30 min (200 ×, scale bar 25 µm) (*n* = 5). D) Representative immunoblots of TGF*β*1, A‐FABP, and HSP90 in culture‐activated A‐FABP KO HSCs treated with different doses of rA‐FABP or vehicle (veh, PBS) for 24 hours. E) The mRNA abundance of *Tgfb1* in the culture‐activated A‐FABP KO HSCs treated with rA‐FABP (2 µg mL^−1^) or vehicle (veh, PBS) for 24 h (*n* = 5). Data are presented as mean ± SD. *P < 0.05, **P < 0.01. Unpaired Mann‐Whitney *U* test was used in (A). Two‐way ANOVA followed by Tukey's test was used in (B). Unpaired Student's *t* test was used in (E).

Next, we determined the target cell type of LSEC‐derived A‐FABP that is responsible for TGF*β*1 production contributing to liver fibrosis. In sham‐operated WT and A‐FABP KO mice, TGF*β*1 mRNA was most abundantly expressed in macrophages while moderately in LSECs and HSCs. BDL significantly induced the mRNA expression of TGF*β*1 in all the isolated cell types with the greatest induction in HSCs (≈55‐fold relative to its sham‐WT cells) (**Figure** **5A**). However, in the absence of A‐FABP, BDL‐induced TGF*β*1 mRNA expression was significantly attenuated in all the tested hepatic cells and the magnitude of reduction in HSCs was the greatest (Figure 5A) indicating that LSEC‐derived A‐FABP is mainly responsible for the regulation of TGF*β*1 transactivation in HSCs in response to BDL.

We then examined if LSEC‐derived A‐FABP is secreted and acts on HSCs to induce TGF*β*1 transactivation. Culture‐activated^[^ ^29^ ^]^ A‐FABP KO HSCs were treated with conditioned media (CM) of LSECs isolated from WT or A‐FABP KO mice after BDL or sham operation (see the procedure in Figure S7 in Supporting Information). The expression of TGF*β*1 was induced significantly in HSCs treated with CM of BDL‐WT‐LSECs but not in those treated with CM of BDL‐KO‐LSECs (Figure 5B). Notably, the presence of A‐FABP was detected in the cell lysate of A‐FABP KO HSCs treated with CM of BDL‐WT‐LSECs and was much more than those treated with CM of Sham‐WT‐LSECs (Figure 5B). Thus, tracing experiment was performed to evaluate if A‐FABP diffuses into HSCs. Fluorescent signal was observed in HSCs treated with Alexa 488‐labeled recombinant mouse A‐FABP protein (rA‐FABP)^[^ ^30^ ^]^ but not in that treated with Alexa Fluor 488‐labeled immunoglobulin G (IgG) or Alexa Fluor 488 dye alone (Figure 5C) indicating exogenous A‐FABP can diffuse into HSCs.

To further confirm the direct role of A‐FABP in exaggerating TGF*β*1 expression, culture‐activated A‐FABP KO HSCs were stimulated with rA‐FABP or its vehicle (Veh, PBS). Treatment with rA‐FABP induced the protein expression of TGF*β*1 in HSCs in a dose‐dependent manner (Figure 5D). The mRNA expression of TGF*β*1 was also markedly upregulated after rA‐FABP treatment (Figure 5E). These data demonstrated that upon BDL, LSEC‐derived A‐FABP releases and diffuses into HSCs to upregulate the transactivation of TGF*β*1 gene.

### 2.6 A‐FABP Stimulates the Transactivation of TGF*β*1 in HSCs by Activating JNK/c‐Jun Pathway

**Figure 6:**
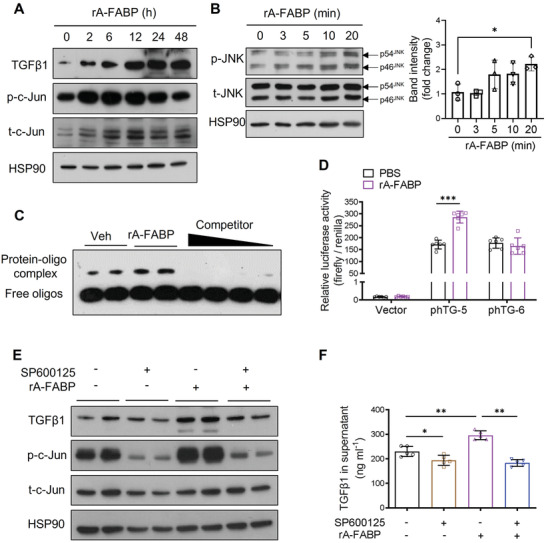
A‐FABP induces TGF*β*1 expression in HSCs by activating JNK/c‐Jun pathway. A,B) Culture‐activated A‐FABP KO HSCs were treated with rA‐FABP (2 µg mL^−1^) for different time durations. Representative immunoblots of A) TGF*β*1, p‐c‐Jun (Ser 63), t‐c‐Jun, and HSP90, and B) p‐JNK (Thr 183/ Tyr 185), t‐JNK, and HSP90 in HSCs. Right panel of (B) is the band intensities of p‐JNK relative to t‐JNK (*n* = 3). C) Culture‐activated A‐FABP KO HSCs were treated with rA‐FABP (2 µg mL^−1^) or vehicle (veh, PBS) for 24 h. Nuclear lysates were subjected to EMSA with the biotin‐labelled probe containing AP‐1 motif of mouse TGF*β*1 promoter with or without 200‐, 150‐, 100‐, and 50‐fold molar excess specific competitors. D) The relative luciferase activities of luciferase‐reporter constructs control vector (pGL3 basic), phTG‐5, or phTG‐6 in HEK293 cells treated with rA‐FABP (2 µg mL^−1^) or vehicle (veh, PBS) for 24 h normalized to renilla values (*n* = 6). E,F) Culture‐activated A‐FABP KO HSCs were pre‐incubated with or without SP600125 (5 × 10^‐6^ m) for 1 h and followed by treatment with rA‐FABP (2 µg mL^−1^) for 24 h. E) Representative immunoblots of TGF*β*1, p‐c‐Jun (Ser 63), t‐c‐Jun, and HSP90 in HSCs. F) Concentration of TGF*β*1 in the conditioned media (CM) of HSCs (*n* = 5). Data are presented as mean ± SD. *P < 0.05, **P < 0.01, ***P < 0.001. One‐way ANOVA followed by Tukey's test was used in (B). Unpaired Student's *t* test was used in (D) and (F).

The transactivation of the TGF*β*1 gene is regulated by the transcription factor AP‐1^[^ ^31^ ^]^ which is mainly composed of c‐Jun and c‐Fos. A‐FABP is part of a finely tuned positive feedback loop with JNK and AP‐1 to exacerbate LPS‐induced inflammatory responses in macrophages.^[^ ^32^ ^]^ Thus, the possibility was explored that A‐FABP induces TGF*β*1 transactivation in HSCs in liver fibrosis through potentiating JNK/c‐Jun signaling. Comparing to WT mice, BDL‐induced phosphorylation of c‐Jun (p‐c‐Jun) in the liver was attenuated significantly in A‐FABP KO mice (Figure S8, Supporting Information). On the contrary, treatment with rA‐FABP induced the phosphorylation of JNK and c‐Jun in HSCs in a time‐dependent manner, which was accompanied by the increased expression of TGF*β*1 (**Figure** **6A**,B). These results implicated that the presence of A‐FABP potentiates the activation of JNK/c‐Jun signaling cascade in HSCs.

Furthermore, electrophoretic mobility shift assay (EMSA) demonstrated the binding of AP‐1 to its cis‐regulatory motifs on TGF*β*1 promoter (biotin‐labeled probe), was enhanced in cultured‐activated HSCs treated with rA‐FABP when compared to those treated with vehicle, while this enhanced binding was abolished with the increasing concentration of specific competitor (the unlabeled oligonucleotides with the same sequence as probe) (Figure 6C). These data indicated that A‐FABP potentiates the JNK/c‐Jun activation thus promotes AP‐1 binding activity and its specific interaction with the AP‐1 cis‐acting motifs on the TGF*β*1 promoter.

We further interrogated if the binding of AP‐1 to the promoter is responsible for the induction of TGF*β*1 expression by A‐FABP. Two luciferase‐reporter constructs, phTG‐5 (‐453/+11) and phTG‐6 (‐323/+11),^[^ ^33^ ^]^ containing truncated mutants of human TGF*β*1 promoter with two AP‐1 binding sites located between nucleotide ‐432 to ‐323 or no AP‐1 binding site, respectively (Figure S9, Supporting Information), and the control vector (pGL‐3 basic vector) were transfected into HEK293 cells and followed by treatment with rA‐FABP or vehicle. The luciferase activities of phTG‐5 and phTG‐6 were similar at their basal levels while treatment with rA‐FABP significantly induced the luciferase activity of phTG‐5, but not that of phTG‐6 (Figure 6D). Taken together with the result of EMSA, these data indicated that the binding of AP‐1 to its cis‐acting site on the TGF*β*1 promoter is essential for the A‐FABP‐mediated TGF*β*1 transactivation.

Furthermore, the induction of mRNA expression (Figure S10, Supporting Information), intracellular protein expression, and secretion of TGF*β*1 in media (Figure 6E,F) upon rA‐FABP treatment were abolished in the presence of JNK specific inhibitor SP600125 (5 × 10^‐6^ m). The effectiveness of SP600125 in suppressing JNK/c‐Jun signaling was confirmed by its inhibitory effect on c‐Jun phosphorylation (Figure 6E). These data indicated that A‐FABP promotes TGF*β*1 transactivation in HSCs by potentiating JNK/c‐Jun signaling.

### 2.7 Treatment with BMS309403 Alleviates BDL‐Induced Liver Fibrosis

**Figure 7:**
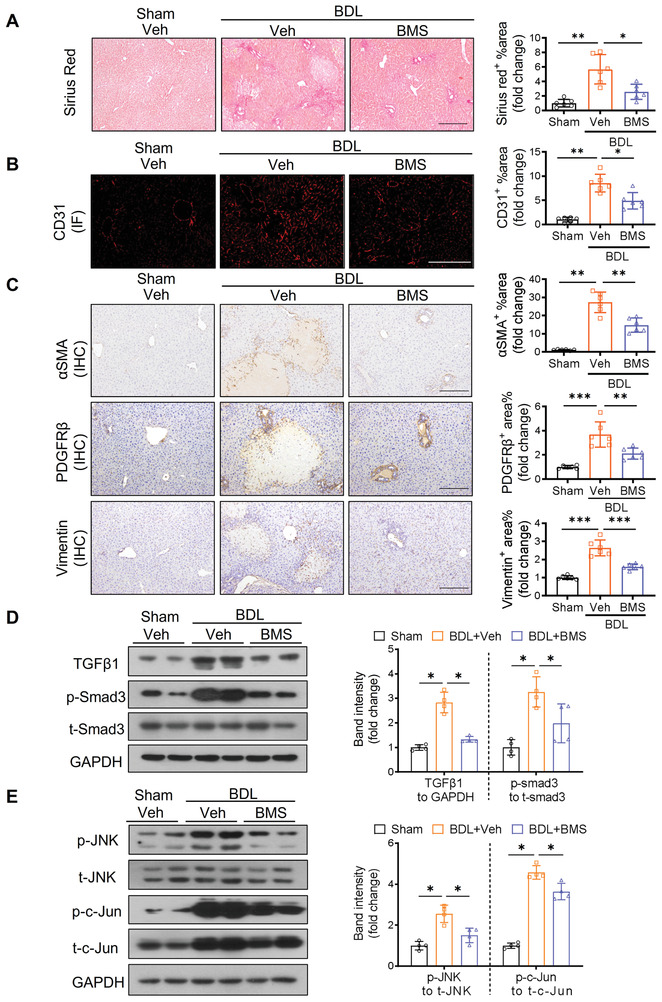
Pharmacological inhibition of A‐FABP alleviates BDL‐induced liver fibrosis. C57BL6/N mice were subjected to BDL or sham operation. From day four after surgery, sham‐operated or BDL‐subjected mice were treated with BMS309403 (BMS, 15 mg kg^−1^ day^−1^) or vehicle (veh, 4% Tween 80) daily by oral gavage for 10 d and sacrificed after 24 h of last gavage (*n* = 6). Representative images of A) Sirius red staining, B) IF staining of CD31, and C) IHC staining of *α*SMA, PDGFR*β*, and vimentin of mouse liver sections (scale bar, 250 µm). Right panels are densitometry analysis of positive area of Sirius red, CD31, *α*SMA, PDGFR*β*, and vimentin, respectively (*n* = 6). Representative immunoblots of D) TGF*β*1, p‐Smad3 (Ser 423/425), t‐Smad3, and GAPDH, and E) p‐JNK (Thr 183/ Tyr 185), t‐JNK, p‐c‐Jun (Ser 63), t‐c‐Jun, and GAPDH in mouse liver. Right panels are the band intensities of various proteins relative to GAPDH or their total protein (*n* = 4). Data are presented as mean ± SD. *P < 0.05, **P < 0.01. Unpaired Student's *t* test was used in (A), (B), and (C). Unpaired Mann‐Whitney *U* test was used in (D) and (E).

We next elucidated the therapeutic effect of selective A‐FABP inhibitor BMS309403 on liver fibrosis. BMS309403 is an orally active small molecule compound specifically designed to compete with fatty acids for the hydrophobic binding pocket of A‐FABP.^[^ ^34^ ^]^ BMS309403 binds to A‐FABP with high affinity (Ki < 2 × 10^‐9^ m) and shows significantly greater selectivity over other FABPs (>100 fold), such as heart FABP (H‐FABP or FABP3, Ki = 250 × 10^‐9^ m) and epidermal FABP (E‐FABP or FABP5, Ki = 350 × 10^‐9^ m).^[^ ^34^ ^]^ With the treatment of BMS309403 (BMS; 15 mg kg^−1^ day^−1^),^[^ ^10^ ^]^ the severity of BDL‐induced liver fibrosis in C57BL6/N mice was significantly alleviated with reduced accumulation of mature collagen, repressed LSEC capillarization indicated by CD31, and attenuated HSC activation indicated by reduced expression of *α*SMA, PDGFR*β*, and vimentin (**Figure** **7A**–C). BDL‐induced TGF*β*1 expression and the subsequent activation of Smad3 signaling were also attenuated after BMS309403 treatment (Figure 7D) and were associating with the impaired JNK/c‐Jun signaling (Figure 7E). These data suggested the beneficial effect of BMS309403 on liver fibrosis is at least partially attributable to its alleviation on the LSEC capillarization and the subsequent HSC activation through its suppression on JNK/c‐Jun pathway thus reducing the expression of BDL‐induced TGF*β*1 and its downstream signaling.

## 3 Discussion

Liver fibrosis is potentially reversible while no FDA‐approved medication is available. Interaction between hepatic nonparenchymal cells was shown contributing to liver fibrosis. Thus, identifying the factor(s) that modulates the cell–cell interaction is of critical importance to develop effective therapeutic strategies for the disease. In the present study, evidence from both genetic ablation and pharmacological inhibition in animals demonstrates, for the first time, that A‐FABP is a key mediator of liver fibrosis by coordinating the crosstalk between LSECs and HSCs to initiate and perpetuate HSC activation, thus exaggerates the production of the fibrogenic cytokine TGF*β*1 from HSCs.

Elevation of A‐FABP in different hepatic cell lineages exerts distinct pathogenic effects and has been implicated in different stages of chronic liver disease.^[^ ^10^, ^11^, ^12^, ^13^ ^]^ A‐FABP instigates the inflammatory response in Kupffer cells and infiltrated macrophages contributing to NASH and cirrhosis^[^ ^10^, ^11^ ^]^ while its elevation in intratumoral HSCs and endothelial cells associates with the development of hepatocellular carcinoma.^[^ ^12^, ^35^ ^]^ The present study reveals an increased expression of A‐FABP, especially in the LSECs, in mouse BDL model of liver fibrosis. LSEC is the most abundant non‐parenchymal cell type in the liver, which is essential for blood filtration and endocytosis.^[^ ^36^ ^]^ Two distinct subsets of LSECs were identified. Type 1 LSECs are CD36^hi^CD32^−^CD14^−^LYVE‐1^−^ and are located in the periportal area (Zone 1) while type 2 LSECs are LYVE‐1^+^CD32^hi^CD14^+^CD54^+^CD36^mid‐lo^ and are distributed in the centrilobular region (Zone 3) and the area between Zone 1 and Zone3 (Zone 2).^[^ ^37^ ^]^ Previous studies revealed that large fenestrae disappear in periportal LSECs (Zone 1) in the fetal period while persist in LSECs in centrilobular zones (Zone 3).^[^ ^38^ ^]^ By using single‐cell transcriptomics, Zone 3 LSECs in cirrhotic mouse liver was found to be most susceptible to capillarization as CD34, a marker of LSEC capillarization, and the genes of extracellular matrix were most upregulated in Zone 3 LSECs among the three clusters of LSECs in Zones 1, 2, and 3.^[^ ^39^ ^]^ In the present study, LSECs (stabilin‐2^+^/CD11b^−^) were sorted and used in the in vitro system. As more than 95% stabilin‐2^+^ LSECs were reported to be LYVE‐1 positive,^[^ ^17^ ^]^ thus the LSECs used in our in vitro studies are mainly type 2 LSECs, which include the Zone 3 LSECs with large fenestrae and exhibited the highest potential to capillarization. Differentiated LSECs, with open fenestrae arranged in sieve plates,^[^ ^36^ ^]^ maintain the quiescent phenotype of HSCs and promote the restoration of activated HSCs to the quiescent stage.^[^ ^26^ ^]^ Upon the stimulation of damage factor, differentiated LSECs undergo capillarization leading to reduced fenestrae and enhanced expression of LSEC activation marker CD31,^[^ ^23^ ^]^ thus losing its suppressive effect on HSC activation and potentiating the fibrogenic events in HSCs and impair the regression of fibrosis.^[^ ^26^ ^]^ Our data showed that A‐FABP deficiency protects against LSEC capillarization in BDL‐induced liver fibrosis in mice while overexpression of A‐FABP in LSECs enhanced the capillarization process which was accompanied by the activation of the Hh signaling pathway that is responsible for LSEC capillarization.^[^ ^24^ ^]^ Most importantly, overexpression of A‐FABP in LSEC impaired its protective effect on HSC activation. Here we identified A‐FABP as a novel factor that mediates LSECs capillarization thus initiates HSC activation.

Numerous factors derived from capillarized LSECs modulate the progression of liver fibrosis.^[^ ^40^ ^]^ Capillarized LSEC‐derived fibronectin, EIIIA, and platelet‐derived growth factor induce HSC activation and motility.^[^ ^41^, ^42^, ^43^ ^]^ Capillarized LSECs also mediate angiocrine signals such as FGFR1‐CXCR4 to promote fibrosis.^[^ ^40^ ^]^ In addition to induce LSEC capillarization, the present study also identified that A‐FABP is a novel LSEC‐derived pro‐fibrotic factor which diffused into HSCs and induced the expression and secretion of TGF*β*1 in a paracrine manner. The paracrine effects of A‐FABP have been associated with the pathogenesis of various diseases. For instance, epicardial adipose tissue‐derived A‐FABP acts on cardiomyocytes and results in heart remodeling and failure.^[^ ^44^ ^]^ A‐FABP also acts on cancer cells to promote their proliferation and aggressiveness.^[^ ^45^ ^]^

TGF*β*1‐Smads signaling is the critical driver of collagen accumulation in fibrotic disease.^[^ ^6^ ^]^ TGF*β*1 is synthesized intracellularly in HSCs as a latent complex, which is released and converted to active form by proteolytic cleavage.^[^ ^6^ ^]^ Active TGF*β*1 acts on the cell surface receptor T*β*Rs complex on HSCs in an autocrine manner to activate Smads signaling and induce the production of TIMPs and collagens contributing to the accumulation of ECM.^[^ ^6^ ^]^ Among the Smad proteins, Smad3 and Smad4 exert profibrotic function while Smad2 and Smad7 are protective in the context of hepatic fibrosis.^[^ ^46^ ^]^ The present study indicated that BDL‐induced TGF*β*1 was associated with the increased expression of T*β*Rs and the subsequent activation of Smad3 signaling in WT mice which were attenuated in the A‐FABP KO mice. Taken together with the evidence that HSCs predominately secrete TIMPs contributing to liver fibrosis^[^ ^47^ ^]^ whereas pharmacological inhibition of A‐FABP attenuates the hepatic expression of TIMP‐1 in diet‐induced obese mice,^[^ ^10^ ^]^ this evidence support the detrimental action of A‐FABP in liver fibrosis is at least partially exerted through its regulation on TGF*β*1 expression.

Activation of JNK signaling pathway is observed in activated HSCs in patients with liver fibrosis^[^ ^48^ ^]^ while blocking JNK activity with SP600125 inhibited HSC activation associating with decreased expression of *α*SMA and reduced HSC proliferation.^[^ ^49^ ^]^ Indeed, A‐FABP promotes the transactivation of TGF*β*1 by potentiating JNK/c‐Jun signaling. A‐FABP deficiency attenuated JNK/c‐Jun activation associated with a reduced hepatic expression of TGF*β*1 in BDL‐subjected mice. On the contrary, recombinant A‐FABP induced JNK/c‐Jun activation accompanied by increased TGF*β*1 production, which was abolished in the presence of JNK inhibitor SP600125. These data were supported by the previous findings showing A‐FABP is required for the full activation of JNK signaling^[^ ^32^ ^]^ and c‐Jun mediates the expression of TGF*β*1.^[^ ^31^ ^]^ A‐FABP has been shown to regulate the expression of various genes through modulating the activities of several transcription factors such as Janus kinase 2, peroxisome proliferator‐activated receptor gamma, and liver X receptor‐alpha either by direct interacting with the transcription factor or inducing ubiquitination and/or subsequent proteasomal degradation.^[^ ^30^ ^]^ Further investigations are warranted to explore how A‐FABP potentiates JNK/c‐Jun signaling. Beside our findings that JNK promotes TGF *β*1 expression, TGF *β*1 also stimulates HSC activation and migration through activating JNK.^[^ ^48^, ^50^ ^]^ Thus, A‐FABP‐JNK‐TGF*β*1 may form a positive feedback loop which becomes a vicious cycle to exaggerate liver fibrosis.

Pharmacological inhibition of A‐FABP by treatment with BMS309403 attenuates BDL‐induced cholestatic liver fibrosis and LPS‐induced acute liver injury and diet‐induced chronic steatohepatitis,^[^ ^10^ ^]^ through suppressing JNK/c‐Jun signaling leading to the attenuation of TGF*β*1/Smad3 signaling in HSCs, and impairment of inflammatory response in Kupffer cells,^[^ ^10^ ^]^ respectively. In addition to the elevated A‐FABP, activation of JNK/c‐Jun signaling is always observed at different stages during the progression of chronic liver diseases in animal and human studies.^[^ ^51^ ^]^ Activation of JNK signaling in myeloid cells exaggerates the development of hepatitis and hepatocellular carcinoma by promoting inflammatory responses.^[^ ^52^ ^]^ In patients with liver fibrosis, sustained JNK activation was found in activated HSCs.^[^ ^48^ ^]^ As A‐FABP is required for the full activation of JNK/c‐Jun signaling,^[^ ^32^ ^]^ therefore, A‐FABP‐JNK/c‐Jun represents a key pathological axis in the progression of liver disease.

It is suggested that direct targeting JNK or TGF*β*1 may not be an effective therapeutic strategy for liver fibrosis as these two signaling molecules exert multifunctional effects on various biological processes including tissue homeostasis, cell proliferation and differentiation, and protein synthesis.^[^ ^6^, ^51^ ^]^ Global knockout mice of TGF*β*1 and JNK‐1/JNK‐2 double knockout mice are embryonic lethal.^[^ ^53^, ^54^ ^]^ On the contrary, treatment with BMS309403 attenuated the BDL‐induced activation of JNK/c‐Jun and TGF*β*1/Smad3 signaling while retained their basal activities. BMS309403 possesses multiple beneficial effects on various metabolic diseases in animal studies.^[^ ^8^ ^]^ Global A‐FABP KO mice are also metabolic healthy.^[^ ^8^ ^]^ The present findings support that inhibition of A‐FABP is an effective and relatively safe therapeutic strategy for liver fibrosis through suppressing the over‐activated JNK/c‐Jun and the subsequent TGF*β*1/Smad3 signaling, which implicates that targeting A‐FABP may be an effective approach against liver fibrosis caused by different etiologies but not only restricted to cholestasis.

Other than A‐FABP, liver fatty acid‐binding protein (L‐FABP, also known as FABP1) is the major isoform of FABPs that highly expressed in the liver, and is suggested to regulate hepatic lipid homeostasis.^[^ ^8^ ^]^ L‐FABP is predominantly expressed in hepatocytes^[^ ^8^ ^]^ but also expressed in quiescent HSCs.^[^ ^55^ ^]^ Global L‐FABP deletion was shown to protect against hepatic steatosis and fibrogenesis in mice fed with trans‐fat fructose (TFF) diet.^[^ ^55^ ^]^ In terms of the cell‐specific function of L‐FABP, albumin‐Cre‐mediated L‐FABP deletion in both hepatocytes and HSCs exhibited protective effect in steatotic model (TFF diet)‐induced mild fibrogenic injury but not in nonsteatotic models (BDL or CCl_4_)‐induced liver fibrosis^[^ ^56^ ^]^ while HSC‐specific L‐FABP deletion did not alter the fibrogenesis in either steatotic model‐ or nonsteatotic model‐induced liver fibrosis.^[^ ^56^ ^]^ These findings suggested that hepatocyte‐derived L‐FABP potentiates lipid‐associated fibrogenic injury. On the contrary, resolution from CCl_4_‐induced fibrosis was impaired in mice with hepatocyte‐ and HSCs‐ L‐FABP deletion but not altered in mice with either hepatocyte‐ or HSC‐alone L‐FABP deletion.^[^ ^56^ ^]^ In the in vitro study, L‐FABP was found to decrease in concomitant with the loss of lipids during HSC activation while its overexpression restored lipid formation and inhibited HSC activation.^[^ ^55^, ^57^ These findings implicated the beneficial effect of L‐FABP in the resolution of liver fibrosis by restoring quiescence in HSC. In clinical studies, the proteomic screen indicated that L‐FABP was induced in patients with simple steatosis while reduced in the progressive form of NASH (fibrosis stage 2–4) when compared to the mild form (fibrosis stage 0–1).^[^ ^58^ ^]^ The paradoxical effect of L‐FABP in hepatic fibrogenesis and reversal from liver fibrosis as well as its protective role in in vitro HSC activation reveals its complexity in hepatic pathogenesis and distinct cell‐specific function. By comparing to L‐FABP and combining previous and present findings,^[^ ^10^, ^11^, ^12^, ^13^, ^35^ ^]^ the pathogenic role of A‐FABP in the whole spectrum of liver disease from NAFLD to hepatocellular carcinoma is more straightforward, which further highlights the specificity and potential of A‐FABP as the therapeutic target in liver diseases among the lipid‐binding proteins.

The present study demonstrated the direct pathophysiological role of A‐FABP in hepatic fibrogenesis using nonsteatotic animal models. However, LSEC capillarization is known to precede fibrogenesis in different kinds of liver diseases including NAFLD and NASH^[^ ^59^ ^]^ and the circulating level of A‐FABP is positively correlated to the fibrosis stages in patients with NASH.^[^ ^13^ ^]^ Thus, the role of A‐FABP in fibrogenesis during the progression of NASH warrants further investigation. Studies on the hepatic localization of A‐FABP using specimen of NASH patients and the correlation of A‐FABP expression to the LSEC capillarization and the severity of liver fibrosis in NASH may provide clinical relevance of the current findings.

## 4 Conclusion

**Figure 8:**
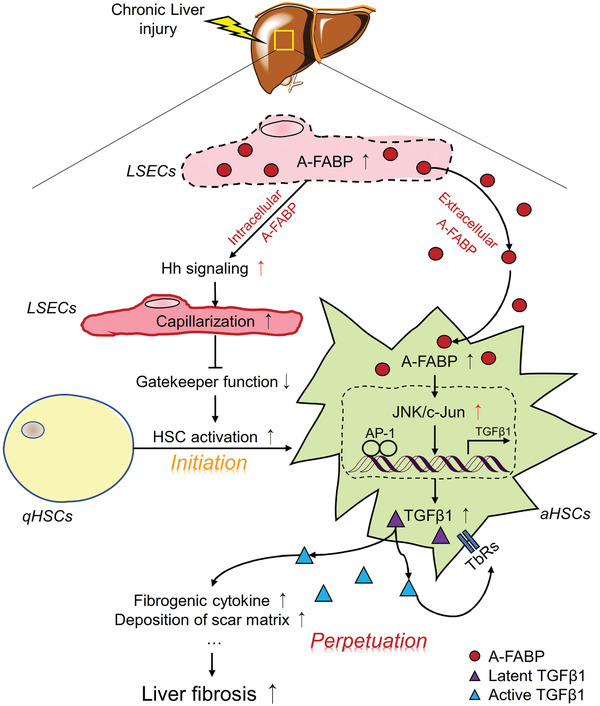
Schematic diagram illustrating the mechanism whereby A‐FABP potentiates liver fibrosis. In response to chronic liver injury, the expression and the subsequent secretion of A‐FABP from LSECs are induced. Elevated A‐FABP potentiates LSEC capillarization through activating Hh signaling pathway thus initiates HSC activation. On the other hand, LSEC‐derived A‐FABP acts in a paracrine manner which diffuses into HSCs and stimulates the transactivation of TGF*β*1 gene through activating the JNK/c‐Jun signaling. Enhanced TGF*β*1 perpetuates HSC activation and facilitates the fibrogenic events such as fibrogenic cytokine expression and deposition of scar matrix, therefore exaggerating liver fibrosis.

In summary, the present study uncovered that A‐FABP potentiates liver fibrosis by enhancing LSEC capillarization and acting as a paracrine factor to exaggerate TGF*β*1 production in HSCs (**Figure** **8**). The expression of A‐FABP in LSECs is elevated in mice with liver fibrosis. Enhancement of intracellular A‐FABP potentiates the LSEC capillarization through inducing Hh signaling leading to initiation of HSC activation. On the other hand, LSEC‐derived A‐FABP releases and acts on HSCs to potentiate the transactivation of TGF*β*1 by activating the JNK/c‐Jun signaling. Increased HSCs‐derived TGF*β*1 further promotes the expression of ECMs in both paracrine and autocrine manner, which perpetuates the HSC activation. Genetic ablation of A‐FABP protects against BDL‐ and CCl_4_‐induced liver fibrosis. Treatment with selective A‐FABP inhibitor also alleviates the severity of BDL‐induced liver injury. These findings define that A‐FABP is a novel therapeutic target of liver fibrosis.

## 5 Experimental Section

The full description of the experimental section is provided in the Supporting Information.

##### Animals and Experimental Models for Liver Fibrosis

A‐FABP KO mice with C57BL/6N background and their WT littermates were generated as previously described.^[^ ^60^ ^]^ Mice were housed in NKP mouse cages with woodchip bedding and placed in the temperature‐controlled facility (23 °C, 12 h light/dark cycle, 60–70% humidity) with free access to water and food. Mice were allocated to their experimental groups according to their genotypes. No randomization of mice was used. The investigators were not blinded to the experimental groups. All the mice were not fasted before the surgery, treatment, blood collection, or sacrifice were carried out. All mouse experiments were conformed to the ARRIVE guidelines (http://www.nc3rs.org.uk/ARRIVEpdf). All experimental protocols were approved by the Committee on the Use of Live Animals in Teaching and Research at the University of Hong Kong (number: 4306‐17).

To induce cholestatic liver fibrosis, 8 week old male A‐FABP KO mice and their WT littermates were subjected to BDL or sham operation for 2 weeks as previously described.^[^ ^61^ ^]^ In brief, ligation of mouse common bile duct was conducted with two surgical knots using 5‐0 suture. Mice received sham operation were served as relative control. To induce carbon tetrachloride (CCl_4_) toxicity‐induced liver fibrosis,^[^ ^62^ ^]^ 8 week old male A‐FABP KO mice and their WT littermates were subjected to intraperitoneal injection of CCl_4_ (0.3 µL g^−1^ body weight, dissolved in olive oil at a ratio of 1:7) (289116, Sigma‐Aldrich, USA) or olive oil as relative control twice a week for eight weeks. Mice were sacrificed three days after the last injection.

To determine the effect of pharmacological inhibition of A‐FABP on liver fibrosis, 8 week old male C57BL/6N mice were subjected to BDL or sham operation for 2 weeks supplemented with treatment of BMS309403 (15 mg kg^−1^ day^−1^ dissolved in 4% Tween 80 in PBS)^[^ ^9^, ^10^ ^]^ (BM0015, Sigma‐Aldrich, WI, USA) or vehicle (4% Tween 80 in PBS) by daily oral gavage from day four after surgery for a total period of ten days. Mice were sacrificed 24 h after the last oral gavage administration.

##### Fractionation and Culture of Primary LSECs, Macrophages, and HSCs

Various primary hepatic cells were isolated from male mouse liver by a combination of pronase‐collagenase digestion, density‐gradient centrifugation, and subsequent purification using magnetic‐activated cell sorting (MACs) or fluorescence‐activated cell sorting (FACs) as previously described.^[^ ^21^, ^22^ ^]^ Isolated primary cells with purity above 95% were further subjected to experiments. The full description is provided in the supplementary experimental section (Supporting Information).

##### Statistical Analysis

All the replicate experiments including in vivo and in vitro experiments were repeated at least two times. All statistical analyses were performed using Prism 8.0 software (GraphPad Software, San Diego, CA, USA). All data are expressed as mean ± SD. The animal sample size for each study was selected on the basis of literature documenting similar well‐characterized experiments.^[^ ^10^, ^63^ ^]^ For mouse experiments, *n* = 6 to 8 for each group, for in vitro experiments, *n*  ≥  3 for each group. No data were excluded from statistical analysis. Data normality was accessed by the Shapiro‐Wilk test. Differences between two groups were evaluated using the Student's *t* test for normal distributions or the Mann‐Whitney *U* test for non‐normal distributions. Differences between multiple groups were compared using ANOVA, followed by the Tukey's test. Details are shown in the figure legends. All statistical tests were two‐tailed. *p*‐values less than 0.05 were considered to indicate statistically significant differences. Pearson's correlation coefficient (PCC) calculated by Image‐J software (NIH, USA) was used to express the colocalization of the two fluorophores in the immunofluorescence costaining experiment. Representation of the *p*‐value was *p < 0.05, **p < 0.01, ***p < 0.001.

## Conflict of Interest

The authors declare no conflict of interest.

## Author Contributions

X.W. and R.H. completed conception and design of this study. X.W. and R.H. drafted the manuscript and prepared the figures. X.W., L.S., Z.Z., J.L., J.Z., and L.Y.C. conducted the experiments. E. S., C.W., D.Y., K.L., A.X., and R.H. edited the manuscript. A.X. and R.H. approved the final version of the manuscript.

## Supporting information

Supporting Information

## Data Availability

The data that support the findings of this study are available from the corresponding author upon reasonable request.
