# Supplementary material for: Adipocyte Fatty Acid Binding Protein Promotes the Onset and Progression of Liver Fibrosis via Mediating the Crosstalk between Liver Sinusoidal Endothelial Cells and Hepatic Stellate Cells
Source: Adv Sci (Weinh). 2021 Mar 27;8(11):2003721. doi: 10.1002/advs.202003721 (PMC8188197; doi:10.1002/advs.202003721)
Supplement: Supplementary file 1 — Supporting Information [file ADVS-8-2003721-s001.pdf]

© 2021 Wiley-VCH GmbH

ADVANCED SCIENCE
Open Access

Supporting Information

for *Adv. Sci.,* DOI: 10.1002/advs.202003721

Adipocyte fatty acid binding protein promotes the onset and progression of liver fibrosis via mediating the crosstalk between liver sinusoidal endothelial cells and hepatic stellate cells

*Dr. Xiaoping Wu, Dr. Lingling Shu, Zixuan Zhang, Dr. Jingjing Li, Jiuyu Zong, Dr. Lai Yee Cheong, Dr. Dewei Ye, Prof. Karen S.L. Lam, Dr. Erfei Song, Prof. Cunchuan Wang, Prof. Aimin Xu, * Dr. Ruby L.C. Hoo*

Adipocyte fatty acid binding protein promotes the onset and progression of liver fibrosis via mediating the crosstalk between liver sinusoidal endothelial cells and hepatic stellate cells

*Dr. Xiaoping Wu, Dr. Lingling Shu, Zixuan Zhang,Dr. Jingjing Li, Jiuyu Zong, Dr. Lai Yee Cheong, Dr. Dewei Ye, Prof. Karen S.L. Lam, Dr. Erfei Song, Prof. Cunchuan Wang, Prof. Aimin Xu, * Dr. Ruby L.C. Hoo*

Supporting information

Supplementary Figures . . . 3
Figure S1 . . . 3
Figure S2 . . . 4
Figure S3 . . . 5
Figure S5 . . . 8
Figure S6 . . . 9
Figure S7 . . . 10
Figure S8 . . . 11
Figure S9 . . . 12
Figure S10 . . . 13
Supplementary Experimental Section . . . 14
Histological analysis of liver . . . 14
Immunohistochemistry (IHC) staining . . . 14
Immunofluorescence (IF) staining . . . 15
Densitometry analysis and colocalization analysis of staining images . . . 16
Fractionation of LSECs, macrophages, and HSCs . . . 16
Primary culture and purity assessment of LSECs . . . 18
Primary culture and purity assessment of HSCs. . . . 18
Generation of adenovirus over-expressing A-FABP and its transduction in LSECs. . . . 19
Scanning electron microscopy (SEM) and quantitative imaging . . . 19
Co-culture of LSECs and HSCs . . . 19
Treatment of HSCs with conditioned media of LSECs. . . . 20
Generation of recombinant A-FABP protein (rA-FABP) . . . 20
Tracing of Alexa Fluor 488-labeled rA-FABP in HSCs . . . 21
Treatment of SP600125 and rA-FABP in HSCs . . . 21
Transfection and Dual Luciferase Assay . . . 21
Immunocytochemistry (ICC) staining . . . 22
Enzyme-linked immunosorbent assay (ELISA) . . . 22
Electrophoretic mobility shift assay (EMSA) . . . 23
Quantitative PCR (qPCR) . . . 23
Immunoblot Analysis . . . 23
Reference . . . 25
Supplementary Table . . . 26
Table S1 Sequence of primers used in the present study. . . . 26

Supplementary Figures

Figure S1

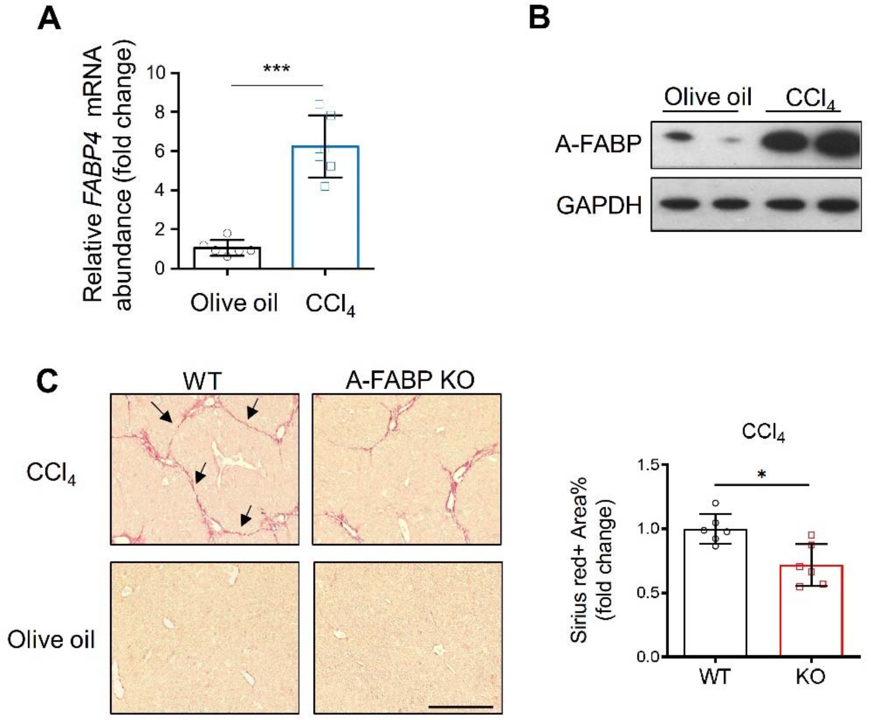

Figure S1. A-FABP deficiency protects against chronic CCl_4 exposure-induced liver fibrosis in mice

Eight-week-old male A-FABP KO mice and their WT littermates were subjected to chronic injections of CCl_4 (0.3μlgram^{-1}) orolive oil (vehicle) for eight weeks (n=6). **(A)** Relative mRNA abundance of hepatic*FABP4* in WT mice (n=6). **(B)** Representative immunoblots of the hepatic expression of A-FABP and GAPDH in WT mice. **(C)** Representative images of Sirius red staining of mouse liver sections (scale bar 250μ m). Black arrow indicates the fibrotic bridging. Right panel is the densitometry analysis ofthe positive areain Sirius red staining of mice in CCl_4 groups and expressed as fold change (n=6). Data are presented as mean ± SD. *$P<0.05$, ***$P<0.001$. Unpaired Student's *t* test was used in (A) and (C).

Figure S2

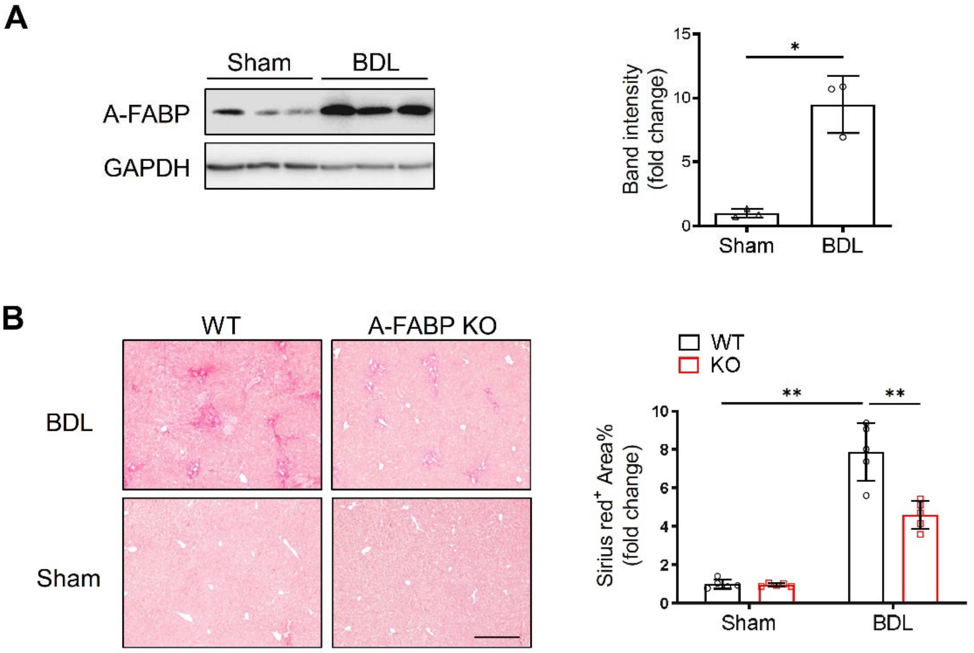

Figure S2. A-FABP deficiency attenuates BDL-induced liver fibrosis in female mice.

Eight-week-old female A-FABP KO mice and their WT littermates were subjected to BDL or sham operation for two weeks (n=5). **(A)** Representative immunoblots of the hepatic expression of A-FABP and GAPDH in mice. Right panel is the band intensity of A-FABP relative to GAPDH (n=3). **(B)** Sirius red staining of mouse liver sections (scale bar 250μm). Right panels are the densitometry analysis of the positive areain Sirius red staining (n=5). Data are presented as mean ± SD. *P<0.05, **P<0.01.Mann-Whitney *U* test was used in (A). Two-way ANOVA followed by Tukey's test was used in (B).

Figure S3

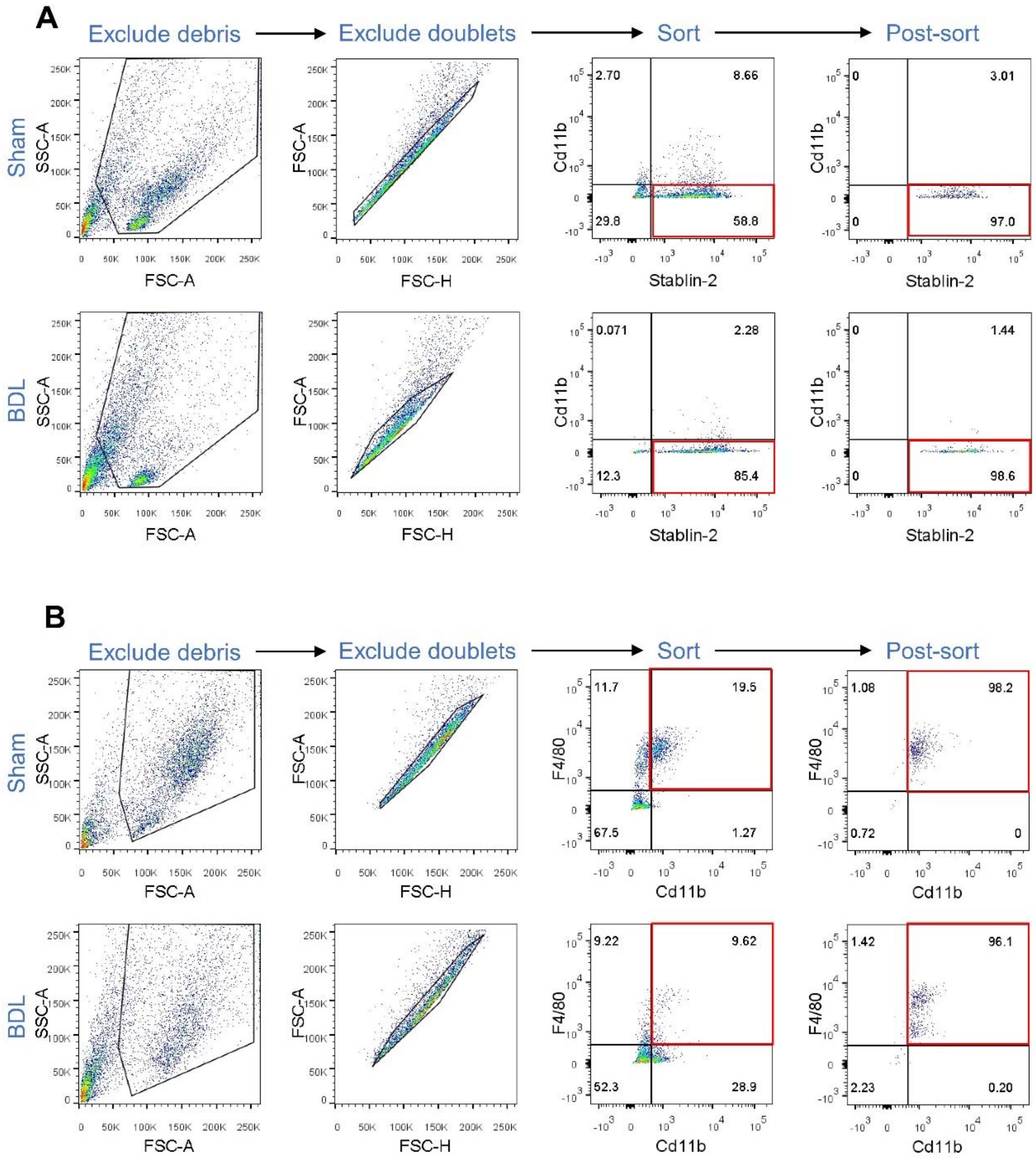
A
Exclude debris
Exclude doublets
Sort
Post-sort
Sham
BDL
SSC-A
FSC-A
FSC-H
Cd11b
Stablin-2
2.70
8.66
29.8
58.8
0
3.01
97.0
0.071
2.28
12.3
85.4
1.44
98.6
B
F4/80
11.7
19.5
67.5
1.27
1.08
98.2
0.72
9.22
9.62
52.3
28.9
1.42
96.1
2.23
0.20

Figure S3 Continuous

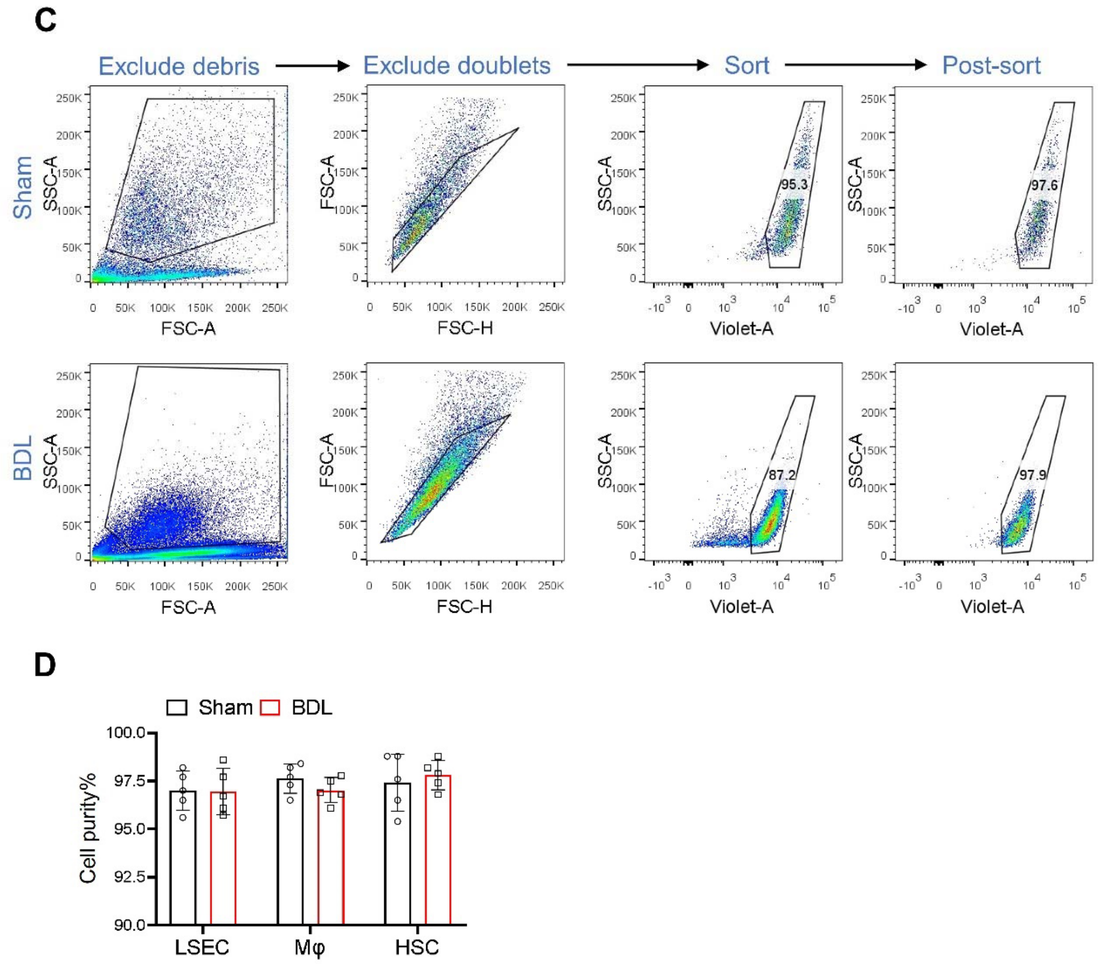

Figure S3. Cell sorting and post-sort analysis of primary non-parenchymal cells subjected to experiments in Figure 2.

(A-C) FACs dot blots showing the sorting gates for LSECs, macrophages, and HSCs isolated from male WT mice subjected to BDL or sham operation for two weeks. Post-sort analysis shows the purity of sorted cells. **(A)** LSECs were defined as Stabilin-2^{+}-cd11b^{-} cells. **(B)** Macrophages were defined as cd11b^{+}-F4/80^{+} cells. **(C)** HSCs were defined as retinoid-based violet-A^{+} cells. **(D)** Cell purity of LSECs, macrophages, and HSCs subjected to the experiments of panel C to E of Figure2 (n=5). Unpaired Student's *t* test or Mann-Whitney *U* test was used in (D).

Figure S4

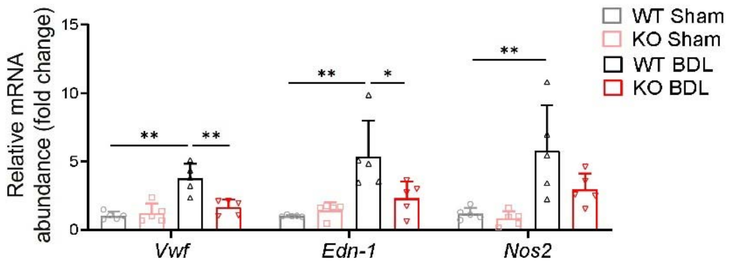

Figure S4. A-FABP deficiency attenuates the BDL-induced LSEC capillarization. Relative mRNA abundance of *Vwf, Edn-1,* and *Nos2* in LSECs isolated from WT and A-FABP KO mice after BDL or sham operation for two weeks (n=5). Data are presented as mean ± SD. *P<0.05, **P<0.01. Two-way ANOVA followed by Tukey's test was used.

Figure S5

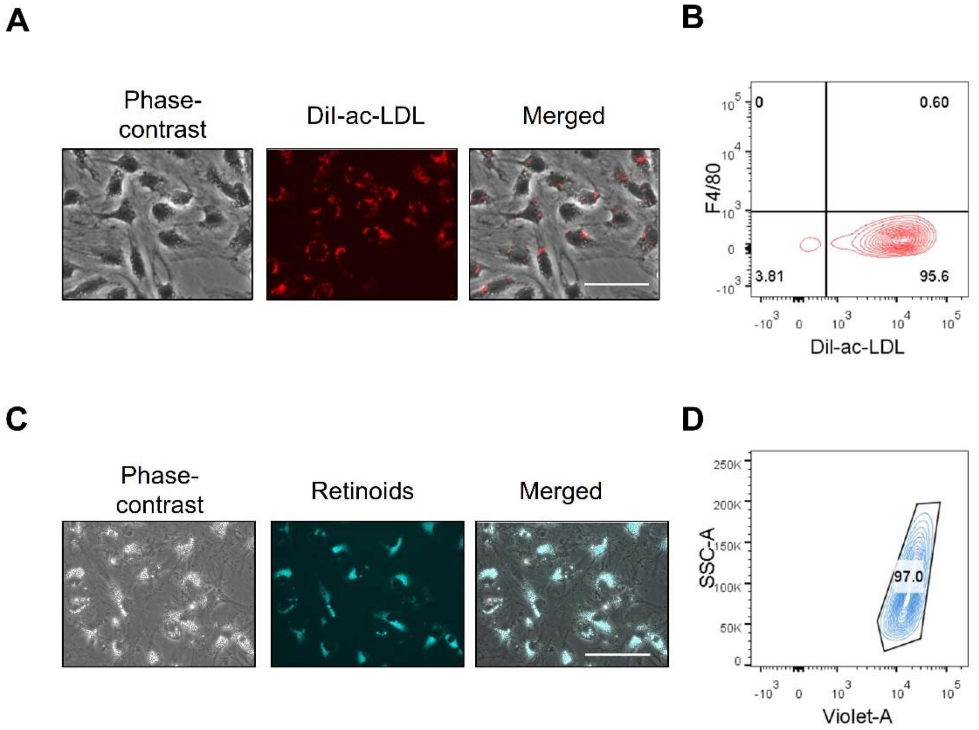

Figure S5. The identity and purity check of LSECs and HSCs isolated from A-FABP KO mice.

(A-B) Following the adherence to cell culture plates, primary LSECs were treated with fluorescentlylabeled dil-ac-LDL (0.5μgml^{-1}) for 12 hours. **(A)** Representative images of cells visualized using phase-contrast microscopy and red fluorescence. Overlap of LSECs and dil-ac-LDL was shown in the merged image (scale bar 100μm). **(B)** Dil-ac-LDL treated cells were labelled with FITC-conjugated anti-mouse F4/80 and processed for flow cytometry. LSECs were defined as dil-ac-LDL^{+}-F4/80^{-} cells, macrophages as dil-ac-LDL^{+}-F4/80^{+} cells and dil-ac-LDL^{-}-F4/80^{+} cells were considered as undefined cells. **(C-D)** Primary A-FABP KO HSCs were cultured for 2 days *in vitro*. **(C)** Representative images of two-day cultured cells visualized using phase-contrast microscopy and blue fluorescence. The overlap of retinoids and lipid droplets was shown in the merged image (scale bar 100μm). **(D)** Cells were subjected to flow cytometry. Retinoid-based violet-A^{+} cells were defined as HSCs.

Figure S6

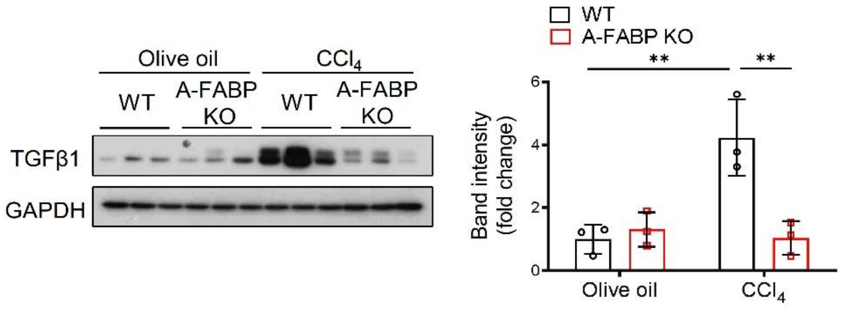

Figure S6. A-FABP deficiency alleviates chronic CCl_4 exposure-induced hepatic TGFβ1 expression in mice

Eight-week-old male A-FABP KO mice and their WT littermates were subjected to chronic injections of CCl_4 (0.3μlgram^{-1}) or olive oil (vehicle) for eight weeks (n=6).Representative immunoblots of hepatic TGFβ1and GAPDH. Right panel is the band intensity of TGFβ1 relative to GAPDH (n=3). Data are presented as mean ± SD. **P<0.01. Two-way ANOVA followed by Tukey's test was used.

Figure S7

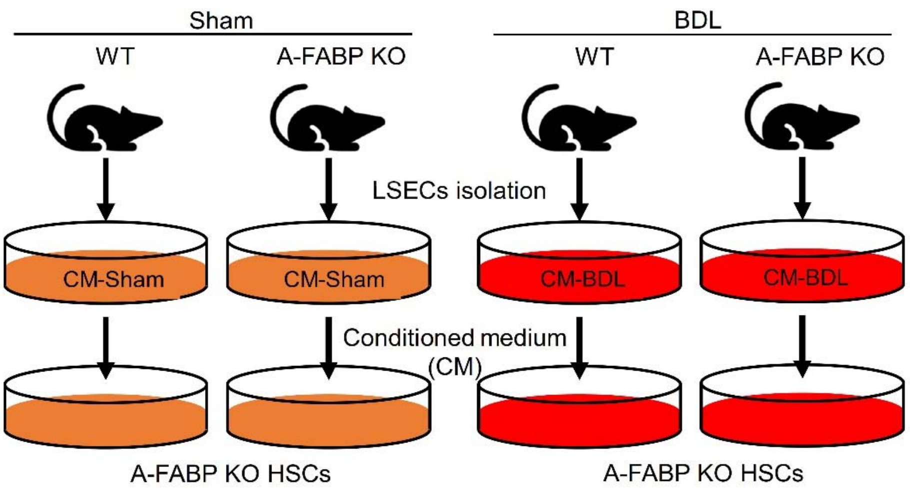

Figure S7. Schematic diagram indicating treatment of A-FABP KO HSCs with conditioned media of primary LSECs isolated from WT or A-FABP KO mice after BDL or sham operation.

Liver sinusoidal endothelial cells (LSECs) were isolated from WT and A-FABP KO mice after BDL or sham operation and cultured for 12 hours. The conditioned media (CM) of LSECs were used for further incubation of culture-activated A-FABP KO HSCs for 24 hours (n=6).

Figure S8

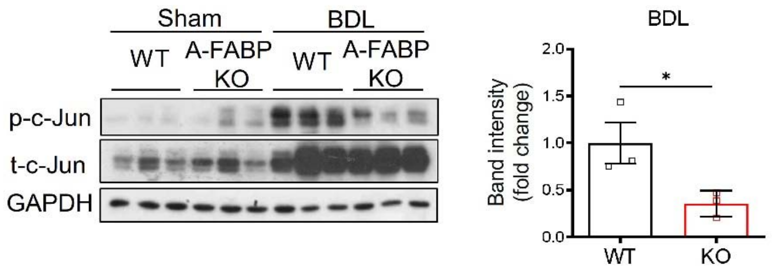

Figure S8. A-FABP deficiency attenuates the phosphorylation of c-Jun in the liver of mice subjected to BDL.

Eight-week-old male A-FABP KO mice and their WT littermates were subjected to BDL or sham operation for two weeks (n=8). Representative immunoblots of the hepatic expression of p-c-Jun (Ser 63), t-c-Jun, and GAPDH. Right panel is the band intensity of p-c-Jun relative to t-c-Jun of mice in BDL group (n=3). Data are presented as mean ± SD. *P<0.05. Mann-Whitney *U* test was used.

Figure S9

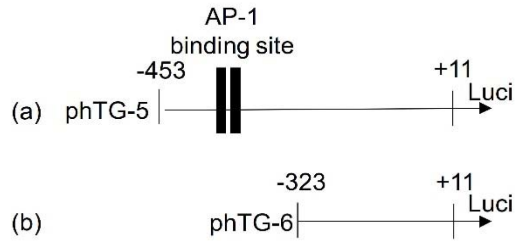

Figure S9. Schematic diagram of TGFβ1 promoter-luciferase reporter constructs. The TGFβ1 promoter-luciferase reporters used in Figure 6D were shown. Two AP-1 binding sites are located between nucleotide -453 to -323 of human TGFβ1 promoter. (a) phTG-5, truncated promoter construct contains two AP-1 binding sites. (b) phTG-6, truncated promoter construct contains no AP-1 binding site.

Figure S10

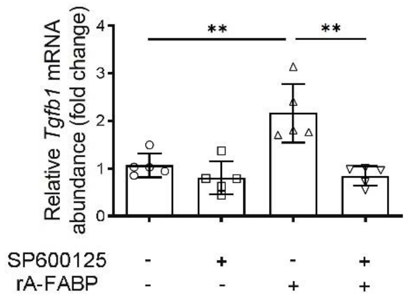

Figure S10. Treatment with SP600125 impairs the stimulating effect of recombinant A-FABP on TGFβ1 mRNA expression in HSCs

Culture-activated A-FABP KO HSCs were pre-incubated SP600125 (5μM) or vehicle (0.01%DMSO) for one hour and followed by treatment with rA-FABP (2μgml^{-1}) or vehicle (PBS, pH7.4) for 24 hours as indicated. Relative mRNA abundance of TGFβ1 in HSCs (n=5). Data are presented as mean ± SD. **P<0.01.Mann-Whitney *U* test was used.

Supplementary Experimental Section

Histological analysis of liver

To access liver fibrosis, Picro Sirius red staining was performed. Liver specimens were fixed in 4% (wv^{-1}) formaldehyde in PBS, then dehydrated and embedded in paraffin as described.[1] The paraffin-embedded mouse liver sections (5μm) were deparaffinized, rehydrated, and stained with Picro Sirius Red Staining Kit (ab150681, Abcam, UK) according to the manufacturer's instructions. The mature collagen positive area in images were analyzed in at least six low-power fields in a blinded fashion in each slide and were quantified using Image-J software (NIH, USA).

Immunohistochemistry (IHC) staining

Paraffin sections of mouse liver specimens were rehydrated and antigen-retrieved with sodium citrate buffer (10mM, pH 6.0) or Tris-EDTA buffer (pH9.0) for 15 minutes in microwaves. The endogenous peroxidase in the tissues was quenched with 3%hydrogen peroxide for 15 minutes at room temperature. Sections were then incubated with 10% fetal bovine serum (FBS) and 3% bovine serum albumin (BSA) in phosphate-buffered saline (PBS) for one hour at room temperature to block non-specific binding. Mouse liver sections were incubated with mouse anti-αSMA antibody (4μgml^{-1}, ab7817, Abcam, UK), anti-PDGFRβ antibody (1μg ml^{-1}, ab32570, Abcam, UK), or anti-vimentin antibody (2μg ml^{-1}, ab92547, Abcam, UK)overnight at 4°C. On the second day, the sections were washed with PBST (PBS containing 0.1% Tween 20) and incubated with respective secondary antibody (4μgml^{-1}, Cell Signaling Technology, USA) for one hour at room temperature. Sections were developed using 3, 3-diaminobenzidine solution (D3939, Sigma Aldrich, USA) and counterstained with hematoxylin to visualize the nucleus. The positive area in images were analyzed in at least six low-power fields in a blinded fashion in each slide and were quantified using Image-J software (NIH, USA).

Immunofluorescence (IF) staining

Paraffin sections and frozen sections ofmouse liver specimens were used to determine the co-localization of A-FAPB and other hepatic cell markersas well as the hepaticcollagen-I and CD31 expression.

To determine the major cellular source of A-FABP in mouse liver, mouse liver sections were subjected to co-staining of A-FABP and the markers of different hepatic cells. Frozen sections used for co-staining of A-FABP and stabilin-2 (marker of LSECs) were fixed with acetone for 10 minutes at -20°C and air dried for 30 minutes. Paraffin sections used for co-staining of A-FABP and F4/80 (marker of macrophages)orαSMA (marker of activated HSCs and smooth muscle cells)were rehydrated and antigen retrieved by incubating sections with EDTA buffer (1mM, pH 8.0) for 15 minutes in microwaves. Both frozen sections and paraffin sections were then washed with PBS for 3 times and incubated with blocking buffer(10% FBS, 3% BSA in PBS) for one hour at room temperature to block non-specific binding. Liver sections were firstly incubated with goat anti-A-FABP antibody (5μgml^{-1}, AF1443, R&D system, USA) overnight at 4°C then followed by washing with PBST and incubation of anti-goat secondary antibody (Alexa Fluor 488, 4μgml^{-1}, A21467, Invitrogen, USA) on the second day. Sections were then washed with PBST and incubated with anti-stabilin-2 antibody (5μg ml^{-1}, D317-3,rat monoclonal, MBL International, USA), anti-F4/80 antibody (5μgml^{-1}, MA516624, rat monoclonal, Invitrogen, USA), or anti-αSMA antibody (4μgml^{-1}, ab7817, mouse monoclonal, Abcam, UK) overnight at 4°C. On the third day, sections were washed with PBST and incubated with respective secondary antibodies (Alexa Fluor 568 or 594, 4μgml^{-1}, Invitrogen, USA).

To determine the hepatic collagen-I accumulation, frozen sections were fixed with acetone for 10 minutes at -20°C and air dried for 30 minutes. Sections were then incubated with blocking buffer for one hour at room temperature and followed by incubation of anti-collagen I antibody (5μg ml-1, ab34710, rabbit polyclonal, Abcam, UK) overnight at 4°C. Sections were then washed with PBST and incubated with anti-rabbit secondary antibody (Alexa Fluor 488, 4μg ml-1, A11008, Invitrogen, USA).

To determine the LSECs capillarization in mouse liver, paraffin sections wererehydrated and antigen retrieved by incubating sections with EDTA buffer (1mM, pH 8.0) for 15 minutes in microwaves. Sections were blocked with blocking buffer for one hour at room temperature then incubated with anti-CD31 antibody (2μg ml^{-1}, ab28364, rabbit polyclonal,Abcam, UK) overnight at 4°C. Sections were then washed

with PBST and incubated with anti-rabbit secondary antibody (Alexa Fluor 568) (4μg ml^{-1}, A11011, Invitrogen, USA).

After the incubation with antibodies and washing, sections were subjected to the mounting process using 4', 6-diamidino-2-phenylindole (DAPI) (62248, Thermo Scientific, USA) to visualize the nucleus. Images were obtained by upright microscope (Eclipse ni, Nikon, Japan) equipped with Precentered Fiber Illuminator (Nikon, Japan).

Densitometry analysis and colocalization analysis of staining images

Densitometry analysis of (1) mature $collagen^{+}$ area in Picro Sirius Red staining, (2) $αSMA^{+}$, $PDGFRβ^{+}$, and $vimentin^{+}$ area in IHC staining, and (3) collagen-I^{+} and $CD31^{+}$area in IF staining was analyzed by Image J software (NIH, USA). Briefly, images were converted to grayscale (8 bit). The positive stained area was segmented by thresholding. The densitometry analysis was represented as the ratio of positive stained area (threshold area) to the total area.

Colocalization analysis was performed using Coloc 2 implements of Image-J software (NIH, USA) to determine the colocalization between immunofluorescencestaining of A-FABP and different hepatic cell markers (stabilin-2, F4/80, and αSMA) in mouse liver sections. Pearson's correlation coefficient (PCC) was used to express the correlation between variables of interest,[2] which is 1 for perfect correlation, 0.5 to 1 for strong correlation, 0.30 to 0.49 for medium correlation, above 0 to 0.29 for small correlation, 0 for no correlation, while below 0 to -1 for anti-correlation.[3]

Fractionation of LSECs, macrophages, and HSCs

Different types of primary hepatic cells were isolated from the liver of WT or A-FABP KO mice subjected to two-week bile duct ligation or sham operation by a combination of pronase-collagenase digestion, density-gradient centrifugation, and subsequent purification using magnetic-activated cell sorting or fluorescence-activated cell sorting (FACs) as previously described.[4,5] Isolated cells with purity above 95% were further subjected to experiments.

Fractionation of LSECs and macrophages were conducted according to previous protocols with modifications.[5]All procedures were conducted at 4°C. In brief, the digested hepatic cell suspension filtered through 70μm cell strainer was centrifuged at

68g for 5 minutes to pellet the parenchymal cells (hepatocytes). The non-parenchymal cells (in supernatant) were pelleted by centrifugation at 600g for 10 minutes and suspended into 17.6% Optiprep (D1556, Sigma-Aldrich, USA). The same amount of 8.2% Optiprep was layered at the top of cell suspension carefully. The 8.2/17.6% Optiprep gradient was then centrifuged at 1400g for 30 minutes with no brake. The cells located in the interface between two density cushions were collected. The cells were then incubated with anti-CD11b microbeads (130049601, Miltenyi Biotec, Germany) and subjected to magnetic-activated cell sorting. The flow-through cells and harvested cd11b$^+$ cells were collected for further purification of LSECs and macrophages, respectively. For LSECs, flow-through cells were stained with anti-stabilin-2 (FITC) antibody (1:100 dilution, D317-A48, MBL International, USA) and anti-cd11b (Pacific blue) antibody (1:50 dilution, 101223, BioLegend, US). For macrophages, harvested cd11b$^+$ cells by magnetic-activated cell sorting were stained with anti-cd11b (Pacific blue) antibody (1:50 dilution, 101223, BioLegend, US) and anti-F4/80 (PE) antibody (1:100 dilution, ab105156, Abcam, UK). FACs was performed by using BD FACSAria SORP (BD bioscience, USA). Voltages were adjusted based on unstained cells and compensation was set using single-stained positive controls for each color. LSECs and macrophages are defined as stabilin-2$^+$-cd11b$^-$ cells and cd11b$^+$-F4/80$^+$, respectively.[4,5] After cell sorting, post-sort analysis was performed to assess the purity of sorted cells. The cell purity was calculated as the ratio of defined cells on the total number of gated single cells.

On the other hand, HSCs were isolated as previously described.[4] In brief, digested hepatic cells was pelleted by centrifugation at 580g for 10 minutes and suspended in Nycodenz medium (1002424, Sigma-Aldrich, USA). Gey's balanced salt solution B (GBSS/B) was layered at the top of the cell suspension. The Nycodenz-GBSS/B gradient was then centrifuged at 1370g for 17 minutes with no brake. The cell pellet located in the interface was HSC fraction. To obtain ultra-purified HSCs, autofluorescence of retinoids was used as cell marker for HSCs in FACs. FACs performing, post-sort analysis, and cell purity calculation were conducted as mentioned above.

To determine the A-FABP secretion, purified LSECs (stabilin-2$^+$-cd11b$^-$), macrophages (cd11b$^+$-F4/80$^+$), and HSCs (retinoid autofluorescence$^+$) were cultured in endothelial cell medium (Cell Biologics, USA), RPMI 1640 Medium (Gibco, USA), or Dulbecco's Modified Eagle Medium (DMEM) (Gibco, USA) containing 1% Penicillin-Streptomycin (P-S) (15140112, Thermo Scientific, USA), respectively. All the isolated cells were incubated in CO_2 incubator (37°C, 5%CO_2, >95% humidity) for

12 hours. The conditioned culture media was collected and subjected to enzyme-linked immunosorbent assay (ELISA) for mouse A-FABP detection (RD291036200R, BioVendor Laboratory Medicine, Czech Republic).

Primary culture and purity assessment of LSECs

LSECs were isolated from eight-week-old male A-FABP KO mouse liver as aforementioned with modifications. After the elimination of $cd11b^+$ cells using magnetic-activated cell sorting, LSECs were purified by cell sorting as mentioned above and long-term selective adherence in culture.[5] Briefly, the cells were suspended in complete endothelial cell medium (Cell Biologics, USA, supplemented with 10mM L-glutamine, 0.75Unitsml^{-1} Heparin sulfate, 1μgml^{-1} hydrocortisone, and 50μgml^{-1} ascorbic acid) containing 1% endothelial cell growth supplements (ECGS, E9640, Sigma-Aldrich, USA), 5% FBS (Gibco, USA), and 1% P-S. Cells were then plated at a density of $4*10^5$cellscm^{-2} in collagen I-coated culture plate then incubated in CO_2 incubator (37 °C, 5% CO_2, >95% humidity) for two hours. After that, the culture medium was removed and the wells were rinsed with calcium-free Dulbecco's Phosphate-Buffered Saline (DPBS) for four times to remove the non-adherent cells. Adherent LSECs were then cultured with complete endothelial cell medium and incubated in CO_2 incubator (37 °C, 5% CO_2, >95% humidity) for the following purity assessment.

LSECs were treated with Dil-Ac-LDL (0.5μgml^{-1}, 022k, Cell applications, USA) for 12 hours in CO_2 incubator (37 °C, 5% CO_2, >95% humidity). The cell morphology and uptake of Dil-Ac-LDL were examined by microscopy with phase contrast and red fluorescence filter. Cells were then subjected to flow cytometry analysis after the staining with anti-F4/80 (FITC) antibody (1:100, 123107, Biolegend, USA). The purity of LSECs was calculated as the ratio of Dil-Ac-LDL$^+$-F4/80$^-$ cells on the total number of gated single cells. The batch of LSECs with purity above 95% were subjected to further experiments.

Primary culture and purity assessment of HSCs.

HSCs were isolated from male A-FABP KO mouse liver as mentioned above. Cells were plated in plastic cell culture plates, cultured with DMEM containing 10% FBS and 1% P-S, and incubated in CO_2 incubator (37 °C, 5% CO_2, >95% humidity). The cell morphology and cellular retinoid storage were examined by microscopy with phase

contrast and blue fluorescence filter using two-day cultured HSCs. The cell purity was accessed by autofluorescence of retinoids using flow cytometry analysis as aforementioned. The batch of primary HSCs with purity above 95% were subjected to experiments. As spontaneously activation of HSCs occurs from 3 days when cultured on plastic in the presence of FBS,[6,7] HSCs harvested on third day in culture were used to study the initiation of HSC activation. HSCs cultured for four days allowing spontaneous activation were used to study the biosynthesis of activated HSCs ("culture-activated HSCs").

Generation of adenovirus over-expressing A-FABP and its transduction in LSECs

Adenoviruses over-expressing A-FABP (Ad-A-FABP) and recombinant adenovirus over-expressing luciferase (Ad-Luci) were generated as previously described.[8,9] Both Ad-A-FABP and Ad-Luci were purified with AdEasy Virus Purification Kit (240245,Strategene, USA) according to the manufacturer's instruction. On the first day in culture, primary A-FABP KO LSECs were infected either with Ad-A-FABP (MOI:50) or Ad-Luci (MOI:50) for 8 hours. The cells were then washed with DPBS and cultured in fresh complete endothelial cell medium for the following 48 hours. Cells were harvested at night of the third day in culture.

Scanning electron microscopy (SEM) and quantitative imaging

Sample preparation was conducted as previously described.[10] LSECs were imaged using Hitachi S3400N VP SEM scanning electron microscope. Porosity, which indicated the percentage of fenestrae on LSEC surface, was measured by the ratio of the open area of fenestrae to the total area of cell surface. The average porosity of each sample was taken from 10 images analyzed by Image J software.

Co-culture of LSECs and HSCs

Primary LSECs and HSCs were co-cultured according to previous protocols with modifications.[11] LSECs and HSCs were isolated from male A-FABP KO mice at the same day. LSECs ($4*10^5$cellscm^{-2}) were plated in collagen I-coated trans-well inserts with 3μm pore size and cultured with complete endothelial cell medium containing 1% endothelial cell growth supplements, 5% fetal bovine serum, and 1% P-S. HSCs ($1.5*10^5$cellscm^{-2}) were plated in the wells of plastic plates and cultured with DMEM

containing 10% FBS and 1% P-S. On the first day in culture, LSECs were infected either with Ad-A-FABP (MOI:50) or Ad-Luci (MOI:50) for 8 hours. After the infection, the culture medium was removed and the LSECs were rinsed once with DPBS. The trans-well inserts were then added into the culture plates of HSCs. LSECs and HSCs were co-cultured for the following 48 hours. Both types of cells were harvested at third day after isolation.

Treatment of HSCs with conditioned media of LSECs

Liver sinusoidal endothelial cells (LSECs) isolated from WT or A-FABP KO mice with two-week BDL or sham operation were purified by FACs as aforementioned and plated at a density of $4*10^5$cellscm^{-2} in six-well plates and cultured with DMEM containing 2% FBS and 1% P-S. After 12 hours, the conditioned media (CM) of different groups of LSECs was collected and centrifuged at 14000 rpm to eliminate the debris. Culture-activated A-FABP KO HSCs were plated at a density of $1.5*10^5$cellscm^{-2} in six-well plates. On the second day, HSCs were then incubated with CM of LSECs for 24 hours. The cell extract of HSCs was then harvested using radioimmunoprecipitation assay (RIPA) bufferand subjected to immunoblot analysis.

Generation of recombinant A-FABP protein (rA-FABP)

Recombinant A-FABP protein (rA-FABP) was generated as previously described.[12] In brief, mouse A-FABP (GenBank BC054426.1) gene was cloned into the His-tag expression vector pDEST17 and then transformed into BL21 Escherichia coli (*E.coli*). The protein expression in *E.coli* was induced by Isopropyl β-D-1-thiogalactopyranoside (500mM; I5502, Sigma-Aldrich) at 37°C for 4 hours. The his-tagged rA-FABP was purified with Ni-NTA resin (88221, Thermo Scientific, USA) and eluted with imidazole (60mM, I5513, Sigma-Aldrich, USA). The eluted protein was subjected to dialysis against 500 times volume with phosphate buffered saline (PBS, pH 7.4). The protein was then filtered through 0.22μm polyethersulfone filter and subjected to endotoxin removal using Pierce High Capacity Endotoxin Removal Spin Columns (88274, Thermo Scientific, USA). The level of endotoxin in the protein was measured by QCL-1000 End point Chromogenic LAL Assays (11670101, Lonza, Switzerland). The purity and identity of rA-FABP were confirmed by SDS–polyacrylamide gel electrophoresis and subsequent Coomassie blue staining or immunoblot analysis. The purified rA-FABP was 5mgml^{-1} with endotoxin below 0.02EU/μg protein.

Tracing of Alexa Fluor 488-labeled rA-FABP in HSCs

To determine the diffusion of rA-FABP into HSCs, labeling of rA-FABP or IgG protein with Alexa Fluor 488 fluorescent dye and the subsequent treatment in HSCs were performed according to previous protocol with modifications.[12]In brief, mouse rA-FABP and non-immune rabbit IgG (Antibody and Immunoassay Service, HKU, HK) were fluorescence-labeled using Alexa Fluor 488 Microscale Protein Labelling Kit (A30006, Invitrogen, USA) according to the manufacturer's instructions. Culture-activated A-FABP KO HSCs were treated with PBS, Alexa Fluor 488 dye, Alexa Fluor 488-labeled rA-FABP (2μgml^{-1}), or Alexa Fluor 488-labeled IgG (2μgml^{-1}) for 30 minutes. Cells were rinsed twice with PBS and fixed with 4% (wv^{-1}) formaldehyde for 10 minutes in room temperature. Cells were then subjected to the counterstain of nucleus using Hoechst 33342 (H3570, Invitrogen, USA) according to the manufacturer's instruction. The brightfield images or fluorescent images of HSCs were obtained using inverted microscope (Nikon Ti2-E Widefield Imaging, Nikon, Japan) with brightfield or FITC and DAPI filter cubes.

Treatment of SP600125 and rA-FABP in HSCs

Inhibition of JNK activation in HSCs was conducted according to previous protocol with modifications.[13] In brief, culture-activated A-FABP KO HSCs were pre-treated with SP600125 (5μM, S5567, Sigma-Aldrich, USA) or vehicle (0.01% DMSO) for one hour. HSCs were then treated with rA-FABP (2μgml^{-1}) or vehicle (PBS, pH7.4) for another 24 hours.

Transfection and Dual Luciferase Assay

The human TGFβ1 promoter luciferase-reporter plasmids, phTG-5 (-453/+11) containing two AP-1 sites, phTG-6 (-323/+11) containing no AP-1 site,[14] and pGL-3 basic (empty control vector) were obtained from VectorBuilder (CA, USA). pRL *Renilla* luciferase control reporter vector was purchased from Promega (WI, USA). For transient transfection, human embryonic kidney (HEK)-293 cells were plated in 24-well plates (5*10^{4} cellscm^{-2}) and incubated with DMEM containing 10% FBS and 1% P-S in CO_2 incubator (37°C, 5% CO2, >95% humidity). On the second day, cells

were co-transfected with 500ng reporter plasmids (phTG-5, phTG-6, or control vector) and 20ng *Renilla* using lipofectamine 3000 transfection system (L3000015, InvitrogenUSA) for 10 hours according to the manufacturer's protocol. Cells were then washed twice with PBS and followed by the treatment of human rA-FABP (2μgml^{-1}, 4504, Biovision, US) or vehicle (PBS, pH7.4) for another 24 hours. Cells were lysed with lysis reagent, and the firefly and *Renilla* luciferase activity were determined using Dual-Luciferase Reporter Assay kit (E1910, Promega, USA) according to the manufacturer's protocol. Firefly luciferase activity was normalized to *Renilla* luciferase activity.

Immunocytochemistry (ICC) staining

After the co-culture experiments of LSECs and HSCs, HSCs plated on coverslips were fixed with 4% (wv^{-1}) formaldehyde in PBS for 10 minutes at room temperature followed by washing with ice-cold PBS for three times. Cells were then subjected to permeabilization by incubation with 0.125% triton X-100 in PBS for 10 minutes and followed by washing with PBS for three times. To block non-specific binding of the antibodies, cells were incubated with 1% BSA, 22.52 mgml^{-1} glycine in PBST for 30 minutes at room temperature. After blocking, cells were incubated with mouse anti-αSMA antibody (4μgml^{-1}, ab7817, Abcam, UK) or rabbit anti-collagen I antibody (4μgml^{-1}, ab34710, Abcam, UK) overnight at 4°C. On the second day, cells were washed with PBS for three times and incubated with anti-mouse secondary antibody (Alexa Fluor 568) or anti-rabbit secondary antibody (Alexa Fluor 488) (4μgml^{-1} Invitrogen, USA) for 1 hour at room temperature. After washing with PBS, the coverslips were subjected to the mounting process using 4', 6-diamidino-2-phenylindole (DAPI) (62248, Thermo Scientific, USA) to visualize the nucleus. Images were obtained by upright microscope (Eclipse ni, Nikon, Japan) equipped with Precentered Fiber Illuminator (Nikon, Japan). The positive area in images were analyzed in at least six low-power fields in a blinded fashion in each coverslip and were quantified using Image-J software (NIH, USA).

Enzyme-linked immunosorbent assay (ELISA)

A-FABP or TGFβ1 concentrations in the conditioned media were measured using mouse A-FABP ELISA kit (RD291036200R, BioVendor Laboratory Medicine, Czech Republic) or mouse TGFβ1 Duoset ELISA kit (DY1679-05, R&D system, USA), respectively.

Electrophoretic mobility shift assay (EMSA)

EMSA was performed by using the LightShift Chemiluminescent EMSA Kit (20148, Thermo Scientific, USA) according to the manufacturer's instruction. The double-stranded oligonucleotides (probe) containing AP-1 motif of mouse TGFβ1 promoter (5'- CTGTGGCCCA*GGGGAGTC*ATGGGAGGGT-3') were labeled at 3'-end using Biotin 3′ End DNA Labeling Kit (89818, Thermo Scientific, USA). The unlabeled oligonucleotides with the same sequence as probe were used as specific competitors.

Quantitative PCR (qPCR)

Total RNA was extracted from cells or tissue with RNAiso Plus (9109, Takara Bio Inc, Japan) according to the manufacturer's instruction. Complementary DNA (cDNA) was prepared using Primescript RT-PCR kit (RR037A, Takara Bio Inc, Japan). Quantitative PCR was performed using SYBR Premix Ex Taq (RR420A, Takara Bio Inc, Japan) and the 7,900 HT PCR machine (Applied Biosystems, USA). The relative mRNA expression of a specific gene was measured with 2−ΔΔCt method and normalized against the house-keeping gene β-actin. All the primers were purchased from Invitrogen (USA), the sequences were listed in Table S1.

Immunoblot Analysis

Total protein was extracted from cells or tissues with radioimmunoprecipitation assay (RIPA) buffer, and the protein concentration was measured by Pierce bicinchoninic acid (BCA) protein assay kit (23225, Thermo Scientific, USA). Protein was separated by electrophoresis using SDS–polyacrylamide gel and transferred to polyvinylidene difluoride membranes. The membranes were blocked with 10% fat-free milk and probed with primary antibodies against A-FABP (0.25 μgml^{-1}, AF-1443) from R&D systems (USA); Gli2 (0.2μg ml^{-1}, sc-271786) CTGF (0.2μgml^{-1}, sc-373936) from Santa Cruz Biotechnology (USA); iNOS (0.2μg ml^{-1}, ab15323), TGFβ1 (0.25μgml^{-1}, ab92486), phospho-Smad-3 (S423+S425, 0.25μgml^{-1}, ab52903), total-Smad-3 (0.25μgml^{-1}, ab40854) and αSMA(1μgml^{-1}, ab7817) from Abcam (UK); phospho-c-Jun (Ser63,1:1000, #9261), total-c-Jun (1:1000, #9165), phospho-JNK (Thr183/Tyr185, 1:1000, #9255), total-JNK(1:1000, #9252), GAPDH (1:2500, #5174), and HSP90

(1:2500, #4874) from Cell Signaling Technology (USA) overnight at 4°C and followed with the incubation of respective peroxidase-conjugated secondary antibodies (1:2500, Cell Signaling Technology, USA) for one hour at room temperature. The protein bands were developed with Amersham ECL western blotting detection reagent (RPN2232, GE Healthcare Life Sciences, UT, USA). The expression of the target protein was normalized against the housekeeping protein (GAPDH for *in vivo* study, HSP90 for *in vitro* study) or the relative total non-phosphorylated protein.

Reference

[1] "Paraffin Processing of Tissue | Protocols Online," can be found under https://www.protocolsonline.com/histology/sample-preparation/paraffin-processing-of-tissue/, **n.d.**

[2] K. W. Dunn, M. M. Kamocka, J. H. McDonald, *Am. J. Physiol. Physiol.* **2011**, DOI 10.1152/ajpcell.00462.2010.

[3] "Pearson Product-Moment Correlation," can be found under https://statistics.laerd.com/statistical-guides/pearson-correlation-coefficient-statistical-guide.php, **n.d.**

[4] I. Mederacke, D. H. Dapito, S. Affò, H. Uchinami, R. F. Schwabe, *Nat. Protoc.* **2015**, *10*, 305.

[5] J. Meyer, S. Lacotte, P. Morel, C. Gonelle-gispert, *Exp. Cell Res.* **2016**, *349*, 291.

[6] S. L. Friedman, F. J. Roll, *Anal. Biochem.* **1987**, DOI 10.1016/0003-2697(87)90673-7.

[7] A. Geerts, R. Vrijsen, J. Rauterberg, A. Burt, P. Schellinck, E. Wisse, *J. Hepatol.* **1989**, DOI 10.1016/0168-8278(89)90076-7.

[8] R. L. C. Hoo, L. Shu, K. K. Y. Cheng, X. Wu, B. Liao, D. Wu, Z. Zhou, A. Xu, *Sci. Rep.* **2017**, *7*, 40657.

[9] K. K. Y. Cheng, K. S. L. Lam, Y. Wang, Y. Huang, D. Carling, D. Wu, C. Wong, A. Xu, *Diabetes* **2007**, DOI 10.2337/db06-1580.

[10] G. Xie, S. S. Choi, W. K. Syn, G. A. Michelotti, M. Swiderska, G. Karaca, I. S. Chan, Y. Chen, A. M. Diehl, *Gut* **2013**, *62*, 299.

[11] L. D. Deleve, X. Wang, Y. Guo, *Hepatology* **2008**, *48*, 920.

[12] L. Shu, R. L. C. Hoo, X. Wu, Y. Pan, I. P. C. Lee, L. Y. Cheong, S. R. Bornstein, X. Rong, J. Guo, A. Xu, *Nat. Commun.* **2017**, *8*, 14147.

[13] X. Hui, H. Li, Z. Zhou, K. S. L. Lam, Y. Xiao, D. Wu, K. Ding, Y. Wang, P. M. Vanhoutte, A. Xu, *J. Biol. Chem.* **2010**, *285*, 10273.

[14] H. W. Bae, A. G. Geiser, D. H. Kim, M. T. Chung, J. K. Burmester, M. B. Sporn, A. B. Roberts, S. Kim, *J. Biol. Chem.* **1988**, *270*, 29460.

Supplementary Table

Table S1Sequence of primers used in the present study.

F: Forward primer; R: Reverse primer

Name	Sequence
Primer *Fabp4* F	5′-CCGCAGACGACAGGA-3′
Primer *Fabp4* R	5′-CTCATGCCCTTTCATAAACT-3′
Primer *Col1a1* F	5′-CAATGGTGAGACGTGGAAAC-3′
Primer *Col1a1* R	5′-GGTTGGGACAGTCCAGTTCT-3′
Primer *Col3a1* F	5′-CTGTAACATGGAAACTGGGGAAA-3′
Primer *Col3a1* R	5′-CCATAGCTGAACTGAAAACCACC-3′
Primer *Acta2* F	5′-GCATCCACGAAACCACCTA-3′
Primer *Acta2* R	5′-CACGAGTAACAAATCAAAGC-3′
Primer *Nos2* F	5′-CAGAGGACCCAGAGACAAGC-3′
Primer *Nos2* R	5′-TGCTGAAACATTTCCTGTGC-3′
Primer *Edn-1* F	5′-AAGTTGGGAAAGAAGTGTAT-3′
Primer *Edn-1* R	5′-AAGATGCCTTGATGCTATT-3′
Primer *Nos3* F	5′-TACGCACCCAGAGCTTTTCT-3′
Primer *Nos3* R	5′-CTTGGTCAACCGAACGAAGT-3′
Primer *Klf2* F	5′-TGCCATCTGTGCGATCGT-3′
Primer *Klf2* R	5′-GGCTACATGTGCCGTTTCATG-3′
Primer *vWF* F	5′-TGTCCAAGGTCTGAAGAAGA-3′
Primer *vWF* R	5′-CAGGACAAACACCACATCCA-3′
Primer *Ptch1* F	5′-CCATACACCAGCCACAGCTTCG-3′
Primer *Ptch1* R	5′-GGAGGCTGGAGTCTGAGAACTG-3′
Primer *Gli2* F	5′-ACCATGCCTACCCAACTCAG-3′
Primer *Gli2* R	5′-CTGCTCCTGTGTCAGTCCAA-3′
Primer *Tgfb1* F	5′-TGAGTGGCTGTCTTTTGACG-3′
Primer *Tgfb1* R	5′-TTCTCTGTGGAGCTGAAGCA-3′

Primer *Ccn2* F	5'-GTGCCAGAAAGCACACTG-3'
Primer *Ccn2* R	5'-CCCCGGTTACACTCCAAA-3'
Primer *Timp-1* F	5’-GGTGTGCACAGTGTTTCCCTGTTT-3’
Primer *Timp-1* R	5’-TCCGTCCACAAACAGTGAGTGTCA-3’
Primer *Pdgfb* F	5′-ATCGCCGAGTGCAAGACGCG-3′
Primer *Pdgfb* R	5′-AAGCACCATTGGCCGTCCGA-3′
Primer *Tgfbr1* F	5′-AAATTGCTCGACGCTGTTCT-3′
Primer *Tgfbr1* R	5′-GGTACAAGATCATAATAAGGCAACTG-3′
Primer *b-actin* F	5′-CGGTTCCGATGCCCTGAGGCTCTT-3′
Primer *b-actin* R	5′-CGTCACACTTCATGATGGAATTGA-3′
